# 36‐Channel Spin and Wavelength Co‐Multiplexed Metasurface Holography by Phase‐Gradient Inverse Design

**DOI:** 10.1002/advs.202504634

**Published:** 2025-05-08

**Authors:** Cherry Park, Youngsun Jeon, Junsuk Rho

**Affiliations:** ^1^ Department of Mechanical Engineering Pohang University of Science and Technology (POSTECH) Pohang 37673 Republic of Korea; ^2^ Department of Chemical Engineering Pohang University of Science and Technology (POSTECH) Pohang 37673 Republic of Korea; ^3^ Department of Electrical Engineering Pohang University of Science and Technology (POSTECH) Pohang 37673 Republic of Korea; ^4^ POSCO‐POSTECH‐RIST Convergence Research Center for Flat Optics and Metaphotonics Pohang 37673 Republic of Korea; ^5^ National Institute of Nanomaterials Technology (NINT) Pohang 37673 Republic of Korea

**Keywords:** high‐capacity metasurfaces, hyperspectral, inverse design, metaholograms, spin and wavelength multiplexing

## Abstract

Metasurface holography has emerged as a versatile tool for manipulating light at subwavelength scales, offering enhanced capabilities in multiplexing high‐resolution holographic images. However, the scalability of channel multiplexing remains a significant challenge. In this paper, a high‐capacity single‐cell metasurface is presented capable of maximizing channels by multiplexing holographic images across both spin and wavelength using a single‐phase map. The achievement of simultaneous multiplexing of left‐ and right‐circular polarization states is detailed across a broad spectral range, from visible to near‐infrared wavelengths, by using a single‐cell metasurface, optimized through an inverse design to minimize loss between the target and output images by automatic differentiation. The phase profile is optimized to encode multiple holographic images without requiring complex meta‐atoms, thereby reducing the fabrication complexity while maintaining high performance. Using this method, two metasurface implementations are demonstrated, an 8‐channel hologram covering both the visible and near‐infrared regions and a 36‐channel hologram operating in the full‐visible spectrum across 18 wavelengths separated by 20‐nm intervals. Furthermore, noise‐related loss functions are incorporated into the optimization process to suppress background noise and minimize inter‐channel crosstalk, resulting in significantly improved image quality and fidelity. This approach offers a reliable solution for further photonic applications such as displays, optical data storage, and information encryption.

## Graphical abstract

A single‐cell metasurface is designed to multiplex holographic images across both spin and wavelength channels using a single‐phase map. By employing inverse design and automatic differentiation, the metasurface achieves 36 channel multiplexing across the visible to near‐infrared spectrum. Crosstalk and background noise are effectively suppressed through loss‐aware optimization, enabling compact and fabrication‐friendly implementations of multi‐channel holography.

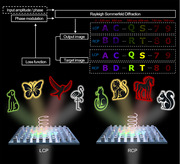

## 1 Introduction

Metaholograms are based on engineered metasurfaces that precisely manipulate light at subwavelength scales and have gained significant attention because of their high‐resolution imaging, multiplexing capabilities, and compact form factors.^[^ ^1^, ^2^, ^3^, ^4^, ^5^, ^6^ ^]^ These metasurfaces enable efficient manipulation of phase, amplitude, and polarization, making them an effective solution for compact, high‐performance optical systems.^[^ ^7^, ^8^, ^9^, ^10^ ^]^ Unlike conventional holography, metaholograms offer the potential for ultra‐compact, lightweight, and efficient devices with applications ranging from optical data storage to 3D displays and secure information encryption.^[^ ^11^, ^12^, ^13^, ^14^, ^15^, ^16^, ^17^ ^]^ Advances in materials like amorphous silicon,^[^ ^18^, ^19^ ^]^ titanium dioxide,^[^ ^20^, ^21^ ^]^ and silicon nitride (SiN_x_)^[^ ^22^, ^23^ ^]^ have further expanded the operational range of metaholograms, allowing for efficient functionality across wavelengths from UV to near‐infrared. Furthermore, the development of multiplexing techniques, including wavelength, polarization, and orbital angular momentum (OAM) multiplexing, has significantly enhanced the functional capacity of metaholograms, enabling simultaneous encoding of multiple holographic images or functionalities.^[^ ^24^, ^25^ ^]^

However, progress in multiplexing has been constrained by inherent limitations in existing methodologies. Most prior wavelength multiplexing studies rely on RGB multiplexing, which encodes holograms across three color channels corresponding to red, green, and blue color channels,^[^ ^26^, ^27^, ^28^, ^29^ ^]^ or polarization multiplexing, which encodes independent images using orthogonal polarization states.^[^ ^30^, ^31^, ^32^, ^33^, ^34^ ^]^ In the case of wavelength multiplexing, conventional approaches frequently employ interleaved structures or supercells, where each structure is dedicated to a specific color channel.^[^ ^35^ ^]^ These methods are often constrained by a limited number of channels. Furthermore, such designs may require a broad spectral range, which inherently limits the overall channel capacity and scalability of the system. To address these challenges, inverse design has been introduced to avoid interleaved metaatom designs. For example, So et al. combined three wavelengths with three z‐direction multiplexing channels to achieve a total of nine channels.^[^ ^36^ ^]^ Similarly, Kim et al. demonstrated hyperspectral holography by encoding holographic images across 10 wavelengths at 30‐nm intervals.^[^ ^37^ ^]^ However, these approaches did not integrate additional multiplexing techniques, thereby limiting their functional capacity. Additionally, studies that multiplex multiple wavelengths using a single‐cell approach exhibit significant limitations in terms of image clarity and noise performance.^[^ ^36^, ^37^, ^38^ ^]^

In the case of spin multiplexing, designs typically rely on two orthogonal polarizations. Separate phase maps are optimized for each polarization.^[^ ^39^ ^]^ In most cases, multiple meta‐atoms are used to combine individually designed phase maps for each polarization into a single metasurface.^[^ ^40^, ^41^ ^]^ This approach implements polarization multiplexing by combining distinct phase maps for each polarization state. However, to date, no studies have demonstrated polarization multiplexing designed through a single optimization process without separately optimizing each polarization state. Traditional forward design methods have played a significant role in achieving multi‐dimensional multiplexing, but they are often constrained by reliance on extensive meta‐libraries and complex physical modeling, which limit scalability and flexibility. To overcome these limitations, recent advancements have introduced deep learning frameworks.^[^ ^42^, ^43^, ^44^, ^45^, ^46^ ^]^ One example is the end‐to‐end framework, which emphasizes the potential to integrate hardware and software optimization for metasurface applications.^[^ ^17^, ^47^, ^48^, ^49^ ^]^ For a metaholography perspective, Yin et al. demonstrated an end‐to‐end inverse design framework that bypasses the need for hologram calculation and reduces dependence on comprehensive meta‐libraries.^[^ ^44^ ^]^ This method successfully implemented a metasurface hologram with up to 12 channels. However, similar to traditional forward designs, this approach still required multiple individual structures. Dependence on such multi‐structure designs necessitates high precision in aligning and fabricating various meta‐atoms to achieve optimal performance, leading to increased manufacturing complexity. Furthermore, the relatively low channel capacity and limited spectral flexibility, constrained to three fundamental wavelengths (RGB), impose additional restrictions on its applicability. To highlight the advancements of our work in comparison to previous studies, we have provided a comparative analysis in Table S1 (Supporting Information).

**Figure 1:**
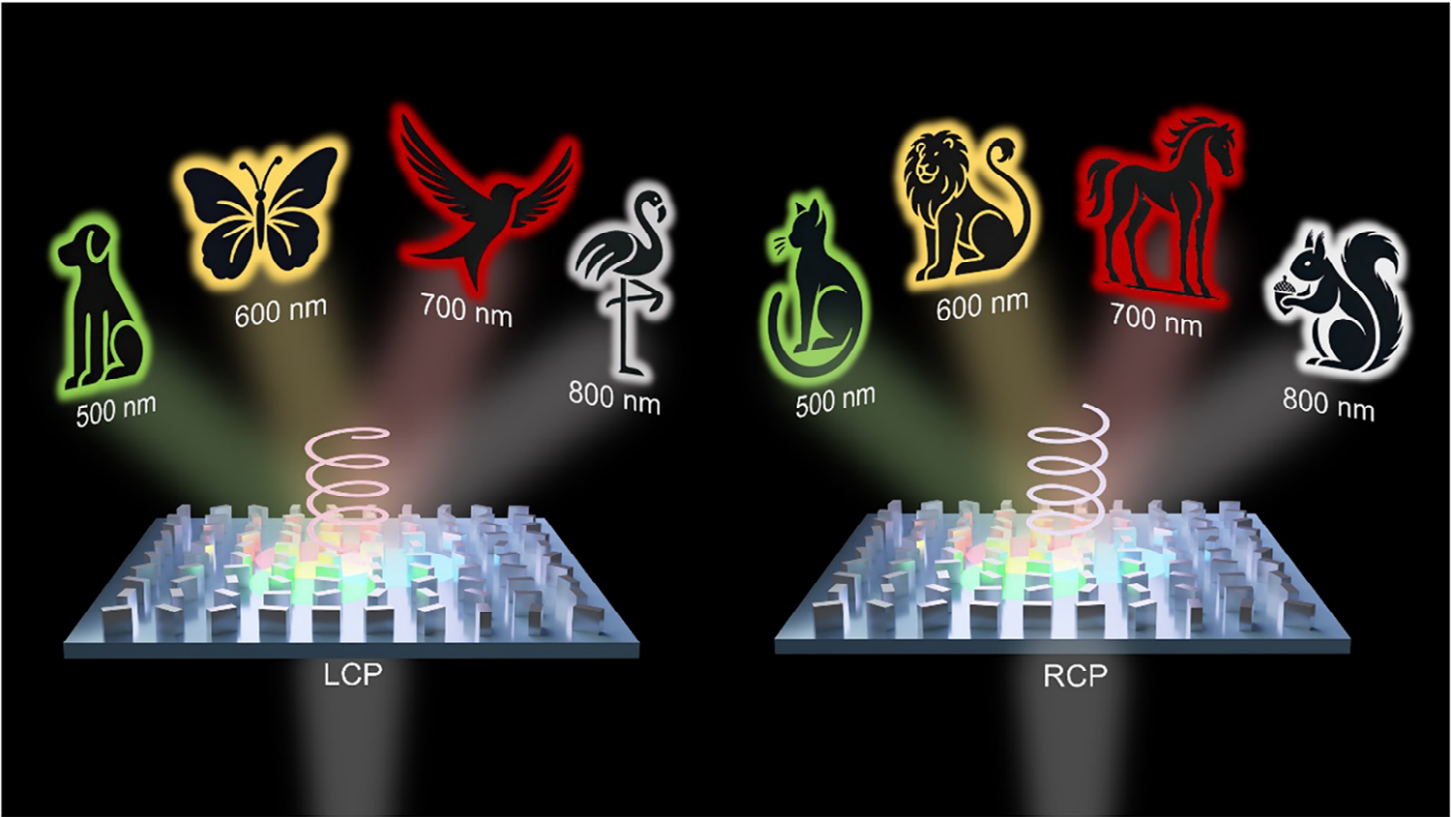
Schematic of a spin‐ and wavelength‐multiplexed metahologram. This hologram encodes multiple holographic images using a single‐cell metasurface, where distinct images appear on the same image plane based on the incident spin states and operating wavelengths.

In this study, we propose an approach that combines hyperspectral and spin multiplexing within a single phase map (**Figure** **1**). Unlike prior studies relying on interleaved designs, separate phase optimizations for each spin state, or restricted spectral ranges, our method simultaneously encodes LCP and RCP information across multiple wavelengths through a single optimization process. By utilizing the Pancharatnam‐Berry (PB) phase of a single meta‐atom within a single phase map, our approach eliminates the need for separate calculations of Φ_𝐿𝐶𝑃_ and Φ_𝑅𝐶𝑃_, thereby reducing both design complexity and fabrication challenges. Moreover, to address the low image fidelity issues reported in previous inverse design‐based single‐cell metasurfaces, our study spatially separates holographic images. Additionally, we employed a narrower spectral range compared to previous studies. To address the potential issue of inter‐wavelength noise, we incorporated two noise suppression loss functions alongside the mean squared error (MSE) loss during the optimization process. This approach optimizes not only the targeted locations but also the surrounding noise regions, allowing for a comprehensive analysis of image quality across the entire metasurface hologram. This methodology enables our design to achieve lower noise levels and higher image fidelity compared to conventional approaches.

To demonstrate the versatility of the proposed approach, we realized two metasurface implementations. The first is an 8‐channel hologram operating across both the visible and near‐infrared (NIR) spectra, enabling broadband operation from 500 to 800 nm for the left‐circular polarization (LCP) and right‐circular polarization (RCP) states. This demonstrates that the proposed design operates effectively over a wide spectral range and confirms that high‐resolution holograms can be achieved across both the visible and NIR regions using only SiN_x_ as a single material, without the need for additional material engineering processes. The second is a 36‐channel hologram optimized for the full‐visible range that encodes 18 wavelengths with two spin states, with a 20‐nm interval between each wavelength. These metasurfaces were fabricated and tested using a supercontinuum laser, demonstrating broadband, high‐capacity holography with minimal crosstalk and precise control over multiplexed images, all appearing on a single image plane. This innovative design provides a scalable and efficient solution for multichannel holography, paving the way for high‐capacity applications such as optical data storage, advanced displays, and information processing.

## 2 Results and Discussion

### 2.1 Concept and Design

Multiple holographic images were encoded onto a single metasurface, with each image determined by wavelength and polarization, thereby distinguishing between the LCP and RCP states. To this end, the phase map was optimized through an inverse design to control the hologram according to the desired wavelength and polarization states. The optimization process minimizes the MSE between the target and output images to ensure similarity within the region of interest (ROI). The ROI is defined based on each target image, and its size varies accordingly. The number of pixels representing the ROI for all target images is provided in Supporting Note S19 (Supporting Information). Additionally, two supplementary loss functions were introduced to further enhance image quality and suppress noise. First, a noise loss ($L_{\mathrm{noise}\;})$ was incorporated to reduce noise outside the ROI by minimizing the output field intensity in the non‐target regions. A target mask (*M*_target_) was used to distinguish the ROI (*M*_target_ = 1) from the background (*M*_target_ = 0), and the noise loss was computed by averaging the output intensity in the non‐target regions. This ensured that noise in the background was effectively suppressed, improving the overall clarity of the reconstructed images. Second, an orthogonality loss minimized crosstalk between adjacent wavelength channels within the same polarization state by reducing the inner product of the output fields of adjacent wavelength channels. This ensures that outputs from neighboring channels remain orthogonal, therby preserving the distinctness of the multiplexed holographic images. The total loss function was designed as a weighted sum of these three loss components:

(1) $$L_{\mathrm{total}\;}=L_{\mathrm{MSE}}+\lambda_{1}\cdot L_{\mathrm{noise}\;}+\lambda_{2}\cdot L_{\mathrm{orthogonality}\;}$$

where, λ_1_ and λ_2_ are the weighting factors that balance the contributions of each loss term. These weights were carefully tuned to optimize the trade‐off between image fidelity and noise suppression (Supporting Note S1, Supporting Information). The Adam optimizer was employed with the metasurface phase as the optimization parameter. The optimizer is represented as an M × N matrix corresponding to the number of meta‐atoms along the horizontal and vertical axes.

**Figure 2:**
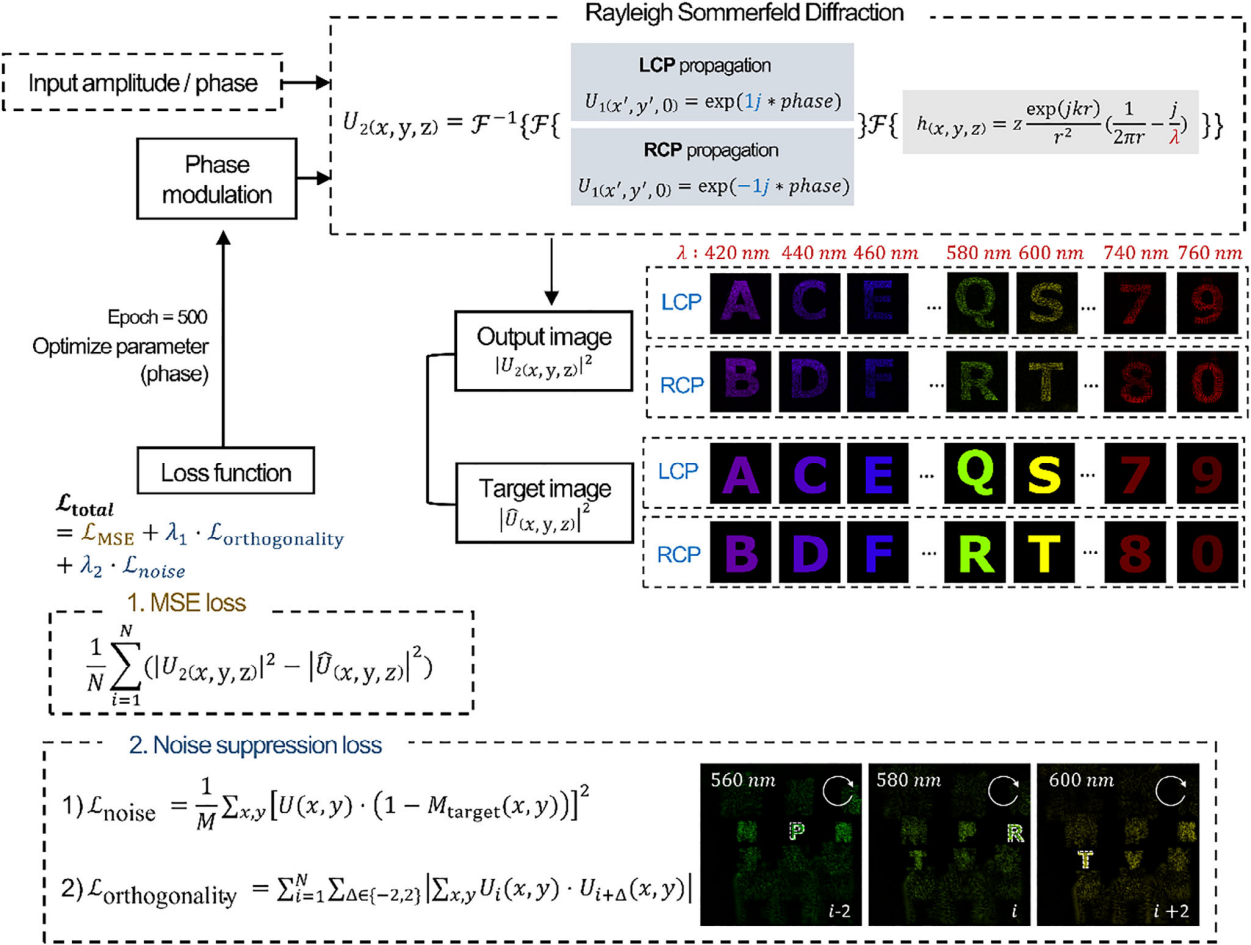
Flowchart of the design process for the spin‐ and wavelength‐multiplexed hologram. Forward wave propagation is computed using Rayleigh–Sommerfeld diffraction, with phase modulation applied to control the LCP and RCP states as well as wavelength. The holographic images corresponding to various wavelengths and spin states are optimized by minimizing the loss between the target and generated images. The phase profile is iteratively refined using gradient based optimization. In addition to the MSE loss, which ensures similarity within the region of interest, additional noise‐related loss terms are incorporated to minimize noise and improve image quality. First, background noise is reduced by calculating values outside the target mask, as indicated by the dotted region (alphabet image) in Noise suppression loss section. Second, to address the significant noise observed between adjacent wavelengths within the same polarization state, an orthogonality loss term is introduced to enhance the orthogonality between channels. The weights for these loss terms are carefully chosen to achieve an optimal trade‐off between image fidelity and noise suppression, ensuring high‐quality reconstruction of the holographic images.

To compute the output images during the optimization, Rayleigh–Sommerfeld diffraction was employed to model wave propagation^[^ ^50^, ^51^ ^]^ (Supporting Note S2, Supporting Information). This method accurately simulates the light–metasurface interaction. As shown in **Figure** **2**, LCP and RCP are encoded simultaneously, while multiple wavelengths are multiplexed to encode various types of holographic information. After forward simulation, the optimization process employed automatic differentiation to update the phase parameters, which were initially set as a zero matrix. Automatic differentiation is a computational technique that automatically computes the derivatives of functions with respect to their input variables. In this case, the input variables are the phase values of the metasurface. Starting from the initial phase, the optimization process iteratively adjusts these values based on the calculated gradients. These gradients are then used to minimize the loss function, which measures the difference between the target holographic image and output image. Through this iterative process, the phase map was refined to improve the accuracy of the hologram images (Figure S4, Supporting Information). PyTorch 1.11.0, a deep‐learning framework with an autograd engine specifically designed for automatic gradient calculation, was utilized to efficiently perform this process.

### 2.2 Meta‐Atom Design

Following the design of the phase map, it is translated into a physical arrangement of meta‐atoms. A locally periodic assumption is applied, where neighboring meta‐atoms are assumed to have similar properties.^[^ ^52^ ^]^ Under this assumption, the interactions between adjacent meta‐atoms are ignored, and each meta‐atom is considered to operate independently. This allows the phase at each point to be physically implemented by matching the meta‐atom to a phase profile based on its optical response, which is simulated under periodic boundary conditions. Although this approach limits the designs to those with slowly varying local responses, it simplifies the overall design and optimization process.

Employing the PB phase eliminates the need for multiple structure types. The PB phase, which arises from the geometric phase shift caused by the rotation of the anisotropic meta‐atoms, enables precise phase control by adjusting the orientation of each meta‐atom.^[^ ^53^ ^]^ By tuning this orientation, opposite phase shifts for the LCP and RCP light can be achieved, allowing for efficient manipulation of the phase profile. The interaction between the incident light and meta‐atom can be modeled using the transmission matrix, as follows:^[^ ^54^ ^]^

(2) $$E=\frac{t_{l}+t_{s}}{2}\left(\begin{array}{c} \;1\;\\ \;\pm i\; \end{array}\right)+\frac{t_{l}-t_{s}}{2}\mathrm{ex}p^{\pm i2\alpha }\left(\begin{array}{c} \;1\;\\ \;\mp i\; \end{array}\right)$$

where *t*_l_ and *t_s_* represent the long‐ and short‐axis transmission coefficients, respectively, and 𝛼 is the rotation angle of the meta‐atom. The term $\left(\begin{array}{c} \;1\;\\ +i\; \end{array}\right)$ corresponds to RCP light, whereas $\left(\begin{array}{c} \;1\;\\ -i\; \end{array}\right)$ represents LCP light. This expression captures the phase modulation for LCP and RCP light caused by the rotation of an anisotropic meta‐atom. By relying on this geometric phase control, we can efficiently encode complex holographic information using a single type of meta‐atom.

**Figure 3:**
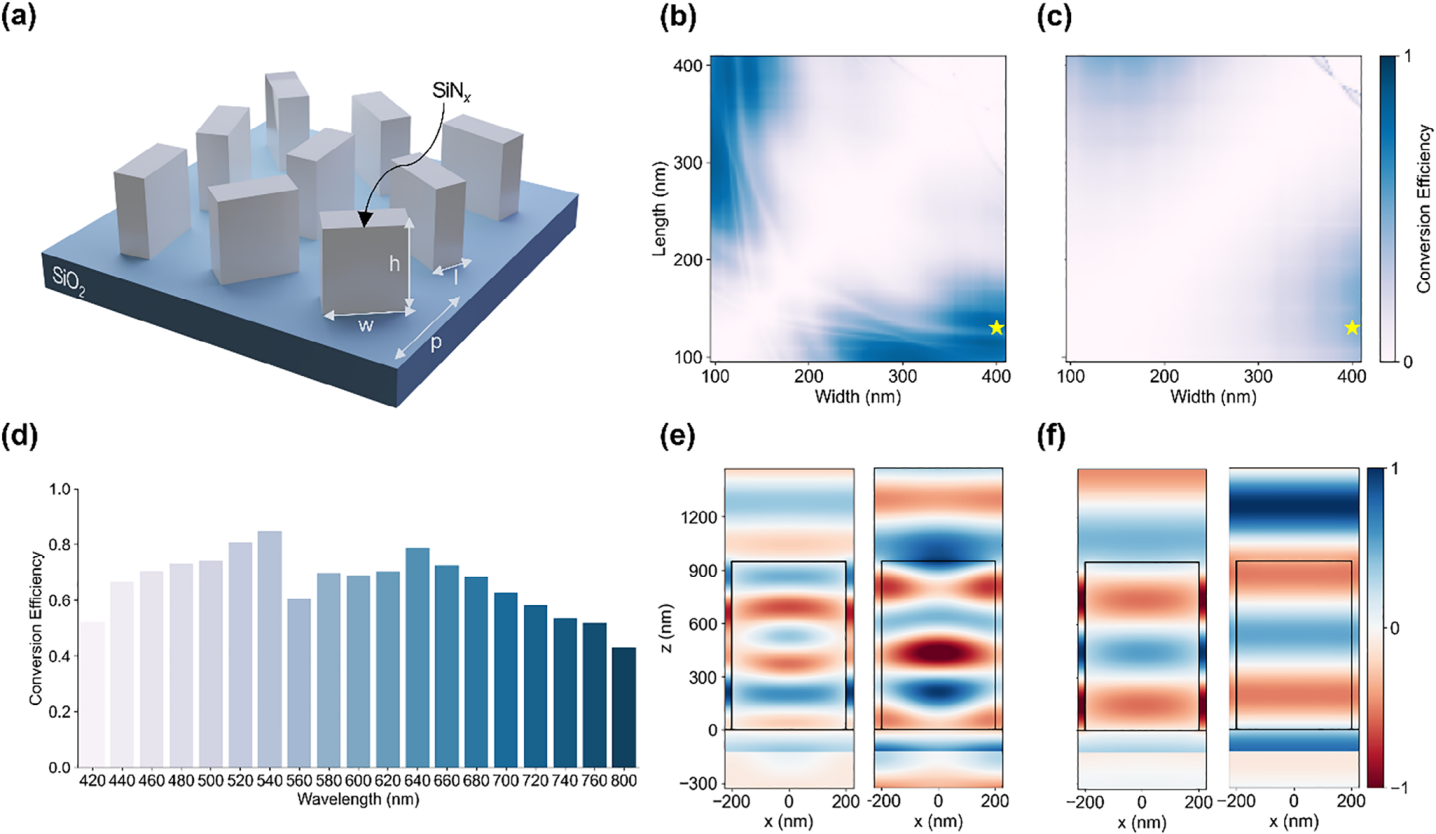
Simulation results of the meta‐atom. a) Schematic of the designed meta‐atom array composed of SiN_x_ structures on an SiO_2_ substrate. Simulated conversion‐efficiency map of the meta‐atom with fixed dimensions of p = 450 nm and h = 950 nm and varying width and length values at wavelengths of b) λ = 500 nm c) and λ = 800 nm. d) Conversion efficiency across 19 wavelengths from 420 to 800 nm. Simulated electric‐field distributions for e) λ = 500 nm f) and λ = 800 nm at x‐polarized (left) and y‐polarized (right) incidences.

SiN_x_ was selected as the material for metasurface implementation owing to its consistent and suitable refractive index and low extinction coefficient across a broad wavelength range, from the visible to the NIR region (**Figure** **3**a; Figure S5, Supporting Information). These properties ensure uniform light manipulation over a broad spectral range, allowing the implementation of low‐loss metaholograms with broadband performance. To achieve a high polarization‐conversion efficiency across the 420–800 nm wavelength range, the optimization process focused on maximizing the total conversion efficiency over the entire spectrum. We simulated the conversion efficiency for a range of meta‐atom dimensions using a rigorous coupled‐wave analysis (RCWA). Simulations were performed across various heights and periods to evaluate their performances. For each combination, the ranges of the length and width were determined based on the fabrication constraints: the minimum size was set at 1/10 of the height to maintain an aspect ratio below 10, and the maximum size was limited to 40 nm smaller than the period to ensure adequate spacing between the meta‐atoms. Based on these simulations, we identified the optimal dimensions of the metasurface as follows: height *h* = 950 nm, period *p* = 450 nm, width *w* = 400 nm, and length *l* = 130 nm. The conversion efficiencies at 500 and 800 nm for varying widths and lengths are shown in Figure 3b,c, respectively, and the efficiencies at other wavelengths are shown in Figure S6 (Supporting Information). The conversion efficiencies of the optimized structure across all the operating wavelengths are shown in Figure 3d. Notably, the minimum efficiency across all wavelengths within the operating range exceeded 0.4.

We verified the function of the meta‐atoms as half‐wave plates by performing finite‐difference time‐domain (FDTD) simulations across the entire operating‐wavelength range. The results for 500 and 800 nm are shown in Figure 3e,f, respectively, and the remaining wavelengths are shown in Figure S7 (Supporting Information). These simulations focused on evaluating the E‐field distribution in the xz plane for both x‐ and y‐polarized incident light. The results indicated a π phase difference between the two polarization states, confirming that the meta‐atoms effectively induce a half‐wave retardation. This behavior was consistently observed across all the targeted wavelengths, validating the meta‐atom as an efficient half‐wave plate within the designed spectral range.

### 2.3 Validation of Spin and Wavelength‐Multiplexed Holograms

**Figure 4:**
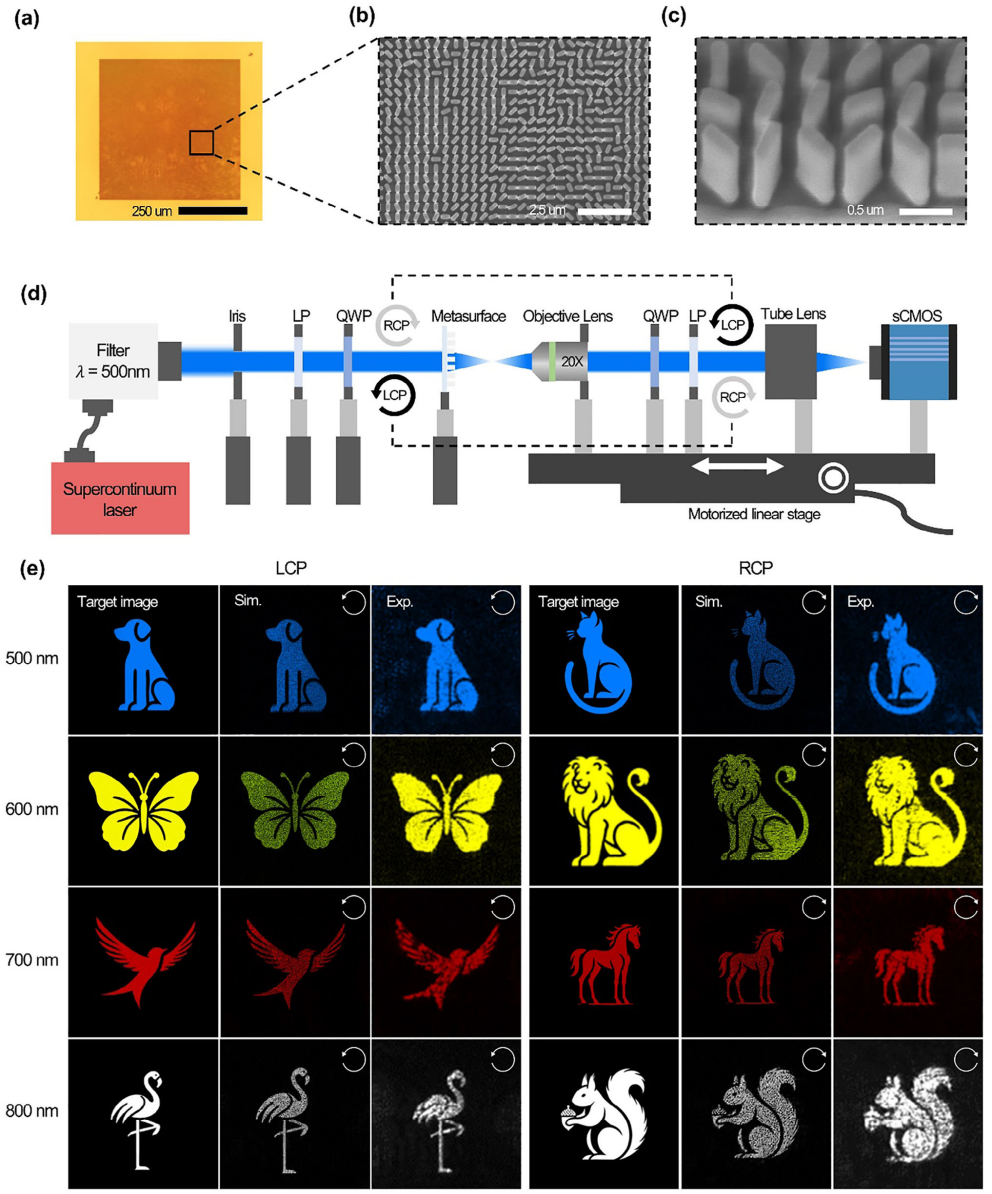
Experimental validation of the fabricated hologram. a) Optical‐microscopy image of the entire fabricated metasurface at × 20 magnification. b) Top view of SEM image at × 10k. c) Tilted view of SEM image at × 50k. d) Optical setup for holographic images under various spin states and wavelengths. (LP: linear polarizer; QWP: quarter‐wave plate) e) Experimentally measured 8‐channel multiplexed hologram images: target images, simulation results, and experimental results for LCP and RCP. Results are shown for wavelengths of 500, 600, 700, and 800 nm.

A metasurface was fabricated through a series of depositions, electron‐beam lithography (EBL), and dry etching to validate the phase map generated by the inverse design experimentally, as illustrated in Figure S8 (Supporting Information). The fabricated metasurface comprised 1100 × 1100 meta‐atoms, covering an area of 495 × 495 µm^2^ (**Figure** **4**a; Figure S9a, Supporting Information). In addition, images of some portions of the fabricated metasurfaces were obtained using scanning electron microscopy (SEM; Regulus 8100, Hitachi) (Figure 4b,c; Figure S9b,c, Supporting Information). Initially, the designed phase covers the full range from 0 to 2π; however, for fabrication simplicity, we utilize only eight phase angles, ranging from 0 to 7π/4 in increments of π/4.

The optical setup for the experimental validation of the fabricated metasurfaces, specifically for spin‐ and wavelength‐multiplexed holograms, is shown in Figure 4d. A supercontinuum laser operating over a wavelength range of 400–2000 nm serves as the light source. We employed wavelengths between 420 and 800 nm, and the individual wavelengths are selected using a tunable bandpass filter. Because the designed hologram exhibits polarization‐dependent behavior, a linear polarizer (LP) and quarter‐wave plate (QWP) were employed to convert the linearly polarized input light into circularly polarized light. As circularly polarized light passes through the metasurface, it is converted to the opposite spin, and the outgoing light is collected by an objective lens. To filter out unconverted light, a second QWP and LP were placed after the objective lens. The light was then transmitted through a tube lens and captured by a scientific complementary metal–oxide–semiconductor (sCMOS) camera. Additionally, the conversion efficiency of the metasurface was measured by determining the ratio of the power of the incident circularly polarized light to the power of the converted circularly polarized light. The incoming circularly polarized light was passed through the metasurface, and only the converted polarization state was filtered using an analyzer and measured using an sCMOS. This process was repeated for all the wavelengths employed in the experiment (Table S4, Supporting Information).

The first sample designed in this study was a hologram that operates in both the visible and NIR regions. It displays different images for LCP and RCP at four distinct wavelengths (500, 600, 700, and 800 nm), resulting in eight multiplexed channels. Each channel was designed to appear at different locations on the same focal plane, specifically at *z* = 900 µm. The simulation and experimental results are shown in Figure 4e, wherein the ROI in the holographic images is magnified. The full simulation and experimental hologram images are shown in Figure S10 (Supporting Information). In the full hologram images, no direct crosstalk occurs between the main images; however, some blurred crosstalk is present. Both the simulation and experimental results show this phenomenon, wherein the designed images appear clean at the target wavelength, but exhibit slight interference at adjacent wavelengths. This blur occurs because the holographic images for each wavelength are not fully isolated, causing leakage or overlap in nearby wavelength regions. This effect is more pronounced at wavelengths close to the target wavelength, leading to the observed crosstalk.

We evaluated the similarity between the target and generated holographic images using three key metrics: the signal‐to‐noise ratio (SNR), peak signal‐to‐noise ratio (PSNR) and structural similarity index (SSIM). The SNR measures the ratio of the average desired image intensity to average intensity of background noise, providing an overall assessment of image clarity. The SNR is defined as:

(3) $$\mathrm{SNR}\;=10log_{10}\left(\frac{I_{\mathrm{signal}\;}}{I_{\mathrm{noise}\;}}\right)$$

The PSNR measures the overall fidelity of the reconstructed image, whereas the SSIM assesses the perceived quality by comparing the luminance, contrast, and structure. The PSNR is calculated using the following formula:

(4) $$\mathrm{PSNR}\;=10\log_{10}\left(\frac{MAX^{2}}{MSE}\right)$$

where *MAX* is the maximum possible pixel value of the image and *MSE* is the mean squared error between the target and output images. SSIM is defined as follows:

(5) $$\mathrm{SSIM}\;=\frac{\left(2\mu_{x}\mu_{y}+C_{1}\right)\left(2\sigma_{xy}+C_{2}\right)}{\left(\mu_{x}^{2}+\mu_{y}^{2}+C_{1}\right)\left(\sigma_{x}^{2}+\sigma_{y}^{2}+C_{2}\right)}$$

where μ_*x*_ and μ_*y*_ are the mean pixel values, $\sigma_{x}^{2}$ and $\sigma_{y}^{2}$ are the variances, σ_*xy*_ is the covariance, and *C*_1_ and *C*_2_ are constants to stabilize the division.

The comparison of SNR, PSNR and SSIM for the reconstructed hologram simulation images, along with the results of continuous (non‐discretized) and 8‐level discretized phase simulations, are shown in Figure S11 (Supporting Information). The continuous‐phase design produces more accurate images and increases the fabrication complexity. In contrast, the 8‐level discretized phase design simplifies the manufacturing process while maintaining comparable image quality, with only a marginal decrease in resolution. This trade‐off between design complexity and practical fabrication constraints is a crucial consideration in metahologram design.

We also compared the PSNR and SSIM values in the ROI, specifically the part of the image containing an animal, between the experimental images captured by the sCMOS and the target images (Figure S12, Supporting Information). For the holograms observed under LCP light, the PSNRs at wavelengths of 500, 600, 700, and 800 nm are 16.33, 12.33, 20.39, and 13.96 dB, respectively, and the SSIM values are 0.8654, 0.7483, 0.9285, and 0.8051, respectively. We conduct the same analysis for the holograms observed under RCP light. The PSNRs for the four wavelengths are 17.70, 16.81, 14.95, and 20.06 dB, respectively, and the SSIM values are 0.9029, 0.8481, 0.8456, and 0.9264, respectively. These results demonstrate that the experimental holograms exhibit a high degree of similarity to the target images. Additionally, the PSNR, SSIM, and SNR values for the full experimental image are provided in Figure S13 (Supporting Information).

**Figure 5:**
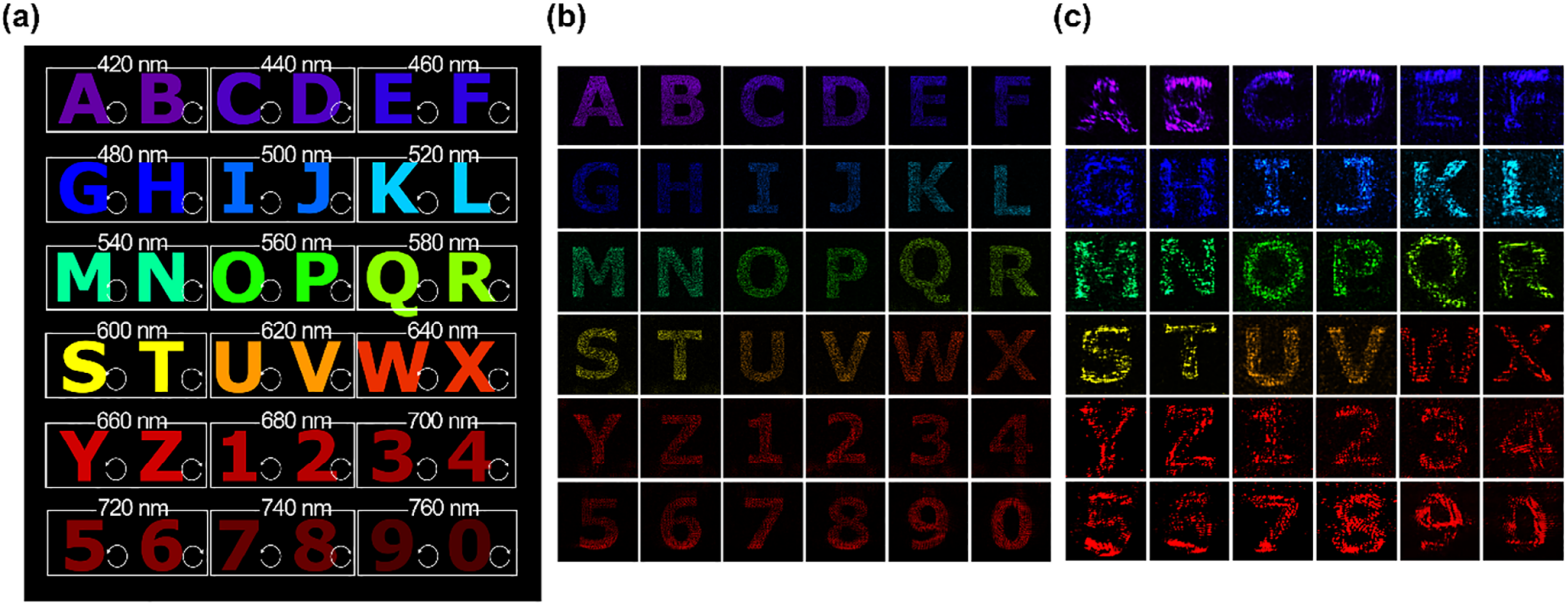
36‐channel multiplexed hologram, operating at 18 wavelengths within the visible spectrum and multiplexed by a spin state to display alphabet and numbers. a) Target images for the 18 wavelengths and two spins. b) Simulated holographic images. c) Experimentally measured holographic images.

The second sample demonstrates multiplexing across 18 wavelengths in the full‐visible range, from 420 to 760 nm, at 20‐nm intervals. By combining wavelength and spin multiplexing, we achieve up to 36 distinct channels, with images appearing at different locations on the same focal plane at z = 900 µm. We confirm the generation of 36 separate images. **Figure** **5** shows a magnified ROI, and the fully simulated images and those captured by the sCMOS sensor are presented in Figure S14 (Supporting Information). The PSNR, SSIM, and SNR values for the full simulation images and experimental images are provided in Figure S15 (Supporting Information). Although crosstalk and blurring occur between adjacent wavelengths, the images at the target wavelengths remain distinguishable. In certain cases, faint images from adjacent wavelengths can appear as noise, such as letters originating from nearby wavelengths. This effect likely results from the phase leakage between closely spaced wavelengths, especially in systems with a high number of multiplexed channels. However, this noise was minimized through optimization that accounted for noise considerations. The proximity of the wavelengths can cause interference between the designed images at adjacent wavelengths. To demonstrate, images are also multiplexed with intervals of 10 and 15 nm for 36 channels. As a result, it is confirmed that as the intervals become narrower, the noise increases significantly, as shown in Figure S16 (Supporting Information). Nonetheless, the main images remain distinct at their intended wavelengths, demonstrating successful multiplexing across all 36 channels. The performance and noise of the hologram are influenced by both the channel count and the spectral interval between adjacent wavelengths. To investigate this relationship, additional simulations were performed. For the 8‐channel hologram, designs with wavelength intervals of 100, 50, 30, 20, and 10 nm were simulated. For the previously studied 20 nm interval, simulations were extended to evaluate holograms with 16 channels, 24 channels, 30 channels, and 48 channels. From these simulations, it was observed that as the spectral intervals decrease, the noise between adjacent channels intensifies, with crosstalk becoming particularly severe when the intervals drop below 20 nm. In terms of channel count, the results indicate no significant degradation in performance for up to 36 channels. However, when the number of channels is increased to 48, there is a noticeable decline in both inter‐channel noise performance and the resolution of the holographic images. The results, including quantitative analyzes, are detailed in Supporting Note S16 (Supporting Information). The differences between the simulation and experimental results are likely due to several factors, including fabrication errors, slight misalignments in setting the focal plane, and angular misalignments during the experimental setup. These factors contribute to deviations in the image quality, leading to observable discrepancies between the designed and experimental holograms. With the same design approach, further channel expansion is possible. By compensating for limitations related to the laser source or material properties, even more channels could be utilized. In this study, simulations verify the multiplexing of a total of 62 holograms across 31 wavelengths ranging from 320 nm to 920 nm, covering regions from the UV to the deeper NIR, as detailed in Supporting Note S17 (Supporting Information).

## 3 Conclusion

In this study, we demonstrated spin‐ and wavelength‐multiplexed holograms by using a single‐cell metasurface. Distinct holographic images were displayed based on the spin states and wavelengths of the incident light, with all the images projected onto the same focal plane. Using an inverse design with automatic differentiation, we optimized a single‐phase map to encode multiple images, Using an inverse design with automatic differentiation, we optimized a single‐phase map to simultaneously encode multiple images across various wavelengths and polarization states through a single optimization process, eliminating the need for independent optimization for each parameter and avoiding the use of complex meta‐atoms.

In addition, we utilized SiN_x_, a material with stable optical properties over a broad wavelength range, which allowed uniform light manipulation. Therefore, a single material was used for uniform light manipulation across all wavelengths. This simplified the fabrication process while ensuring high‐capacity multiplexing and achieving high‐resolution image reconstruction. Using spin and wavelength multiplexing, we extended the number of channels to 36 over a broad range of wavelengths. One of the main advantages of the proposed approach is its scalability, which makes it suitable for high‐capacity applications. Additionally, it allows for encoding large amounts of information while maintaining image fidelity. To achieve this, we introduced an optimization process that spatially separates images and minimizes inter‐wavelength noise between adjacent channels. Our findings may contribute significantly to the design and implementation of metasurface‐based holograms by optimizing a single‐phase map and successfully achieving multiplexing across both the spin and wavelength. This method presents new possibilities for applications in optical data storage, 3D displays, and secure information encryption, for which efficient and scalable holographic systems are in high demand. Future developments can explore the integration of additional degrees of freedom, such as OAM modes, to significantly expand the number of multiplexed channels. By leveraging the orthogonality of OAM modes, numerous distinct channels can be encoded on a single metasurface. In addition, leveraging distinct deep‐learning techniques for engineering crosstalk and noise in holographic images may enable noise‐free holograms across multiple channels. Overcoming material limitations may further allow the implementation of multifunctional metaholograms on a single metasurface spanning the UV–NIR region. The integration of these advanced multiplexing strategies will pave the way for the development of next‐generation holographic systems capable of handling more complex and high‐capacity applications in various fields.

## 4 Experimental Section

### Numerical Simulation

The simulations used in this study include an in‐house RCWA code, which is used to calculate the conversion efficiency of the meta‐atoms, the commercial FDTD solver from Lumerical Inc., ANSYS, was employed to quantify the half‐wave plate‐like behavior of the meta‐atoms.

### Fabrication Method

A fused silica (SiO_2_) substrate was selected for its transparency and integrity for the subsequent fabrication processes. Initially, a 950‐nm thick SiN_x_ film was deposited on the cleaned substrate using plasma‐enhanced chemical‐vapor deposition (HiDep‐SC, BMR Technology). A positive‐tone photoresist (ZEP‐520A, Zeon) was spin‐coated and soft‐baked for the subsequent EBL (ELS‐7800, Elionix). The desired pattern was then achieved by adjusting the electron‐beam dose and time of cold development (ZED‐50, Zeon). A 110‐nm thick Cr layer was deposited on the patterned photoresist through electron‐beam evaporation (KVE‐ENS4004, KVT). A Cr etching mask was deposited on the SiN_x_ film using a lift‐off process. Dry etching (TEL, DRM85DD) was applied to etch SiN_x_. Finally, the remaining Cr mask is removed by immersing the sample in a Cr etchant (ETCR‐400, APCT).

### Optical Measurement

A supercontinuum laser (SuperK FIANIUM, NKT Photonics) was used as the light source, along with a bandpass filter (LLTF contrast, NKT Photonics) to select specific wavelengths. The beam was passed through an iris to match its size. An LP (LPVISE050‐A, Thorlabs, USA) and QWP (AQWP05M‐600, Thorlabs) were used to produce the LCP beam. After passing through the metasurface, the light was collected using a 20x objective lens and analyzed with a QWP, LP, and tube lens (TTL180‐A, Thorlabs). The final holographic images were captured using an sCMOS camera (pco.panda 4.2 bi UV sCMOS camera, Excelitas Technologies).

## Conflict of Interest

The authors declare no conflict of interest.

## Author Contributions

C.P. and Y.J. contributed equally to this work. J.R. conceived the idea and initiated the project. C.P. designed the whole experiments. C.P. conducted the numerical simulations and performed the inverse design for phase map calculations. Y.J. fabricated the devices. C.P. and Y.J. carried out the experiments, including experimental characterizations and data analyses. C.P. and Y.J. mainly wrote the manuscript. All authors confirmed the final manuscript. J.R. guided the entire work.

## Supporting information



Supporting Information

## Data Availability

The data that support the findings of this study are available from the corresponding author upon reasonable request.
