# Supplementary material for: 36‐Channel Spin and Wavelength Co‐Multiplexed Metasurface Holography by Phase‐Gradient Inverse Design
Source: Adv Sci (Weinh). 2025 May 8;12(28):2504634. doi: 10.1002/advs.202504634 (PMC12302594; doi:10.1002/advs.202504634)
Supplement: Supplementary file 1 — Supporting Information [file ADVS-12-2504634-s001.pdf]

ADVANCED SCIENCE
Open Access

Supporting Information

for *Adv. Sci.*, DOI 10.1002/advs.202504634

36-Channel Spin and Wavelength Co-Multiplexed Metasurface Holography by Phase-Gradient Inverse Design

*Cherry Park, Youngsun Jeon and Junsuk Rho**

Supporting Information

36-channel spin and wavelength co-multiplexed metasurface holography by phase-gradient inverse design

Cherry Park[1†], *Youngsun Jeon*[1†], *Junsuk Rho*[1,2,3,4,5*]

C. Park, Y. Jeon, Prof. J. Rho
[1]Department of Mechanical Engineering, Pohang University of Science and Technology (POSTECH), Pohang 37673, Republic of Korea
E-mail: jsrho@postech.ac.kr

Prof. J. Rho
[2]Department of Chemical Engineering, Pohang University of Science and Technology (POSTECH), Pohang 37673, Republic of Korea
[3]Department of Electrical Engineering, Pohang University of Science and Technology (POSTECH), Pohang 37673, Republic of Korea
[4]POSCO-POSTECH-RIST Convergence Research Center for Flat Optics and Metaphotonics, Pohang 37673, Republic of Korea
[5]National Institute of Nanomaterials Technology (NINT), Pohang, 37673 Republic of Korea

Supporting Note 1. Detailed analysis of loss function and impact on holographic image fidelity

(a)

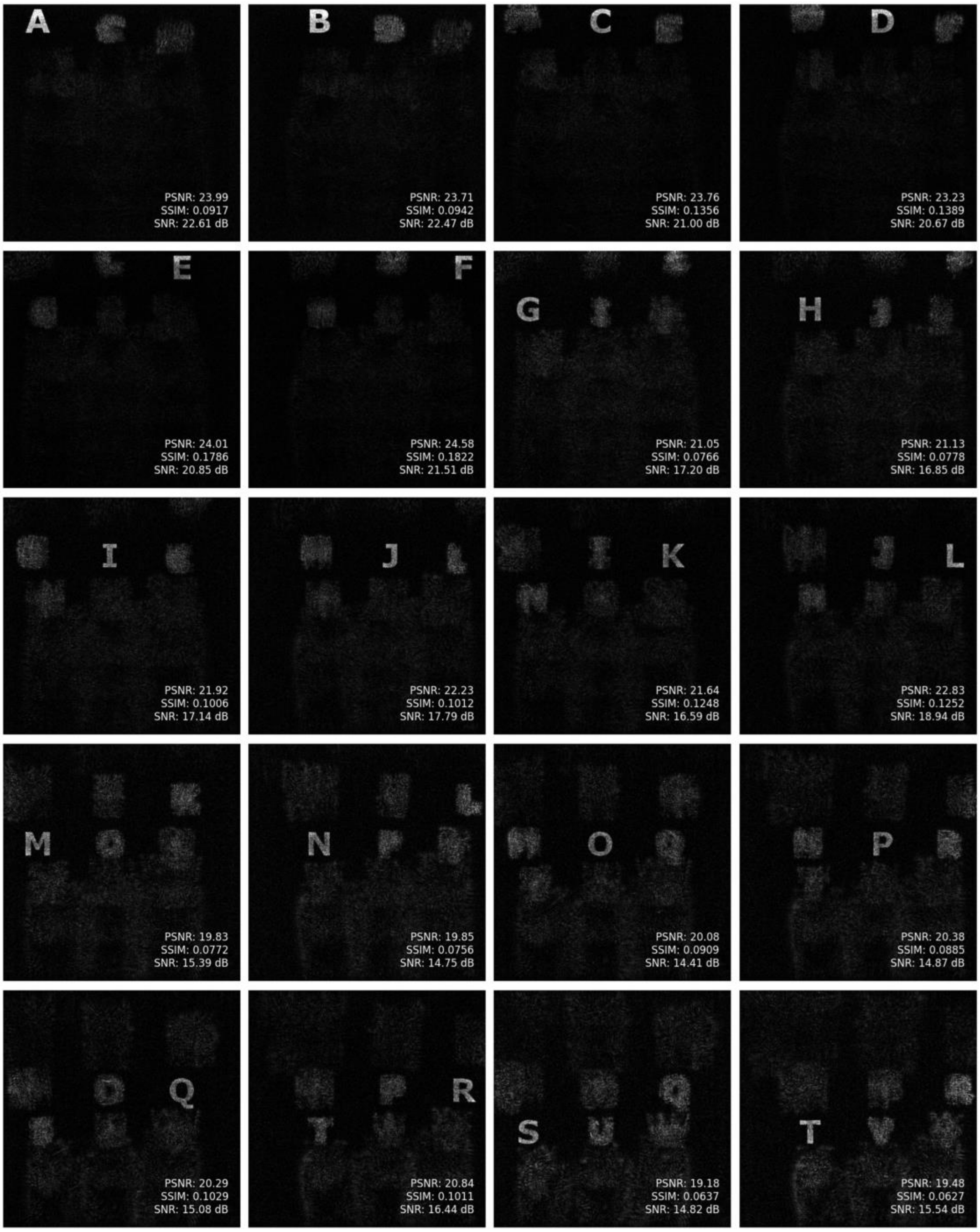

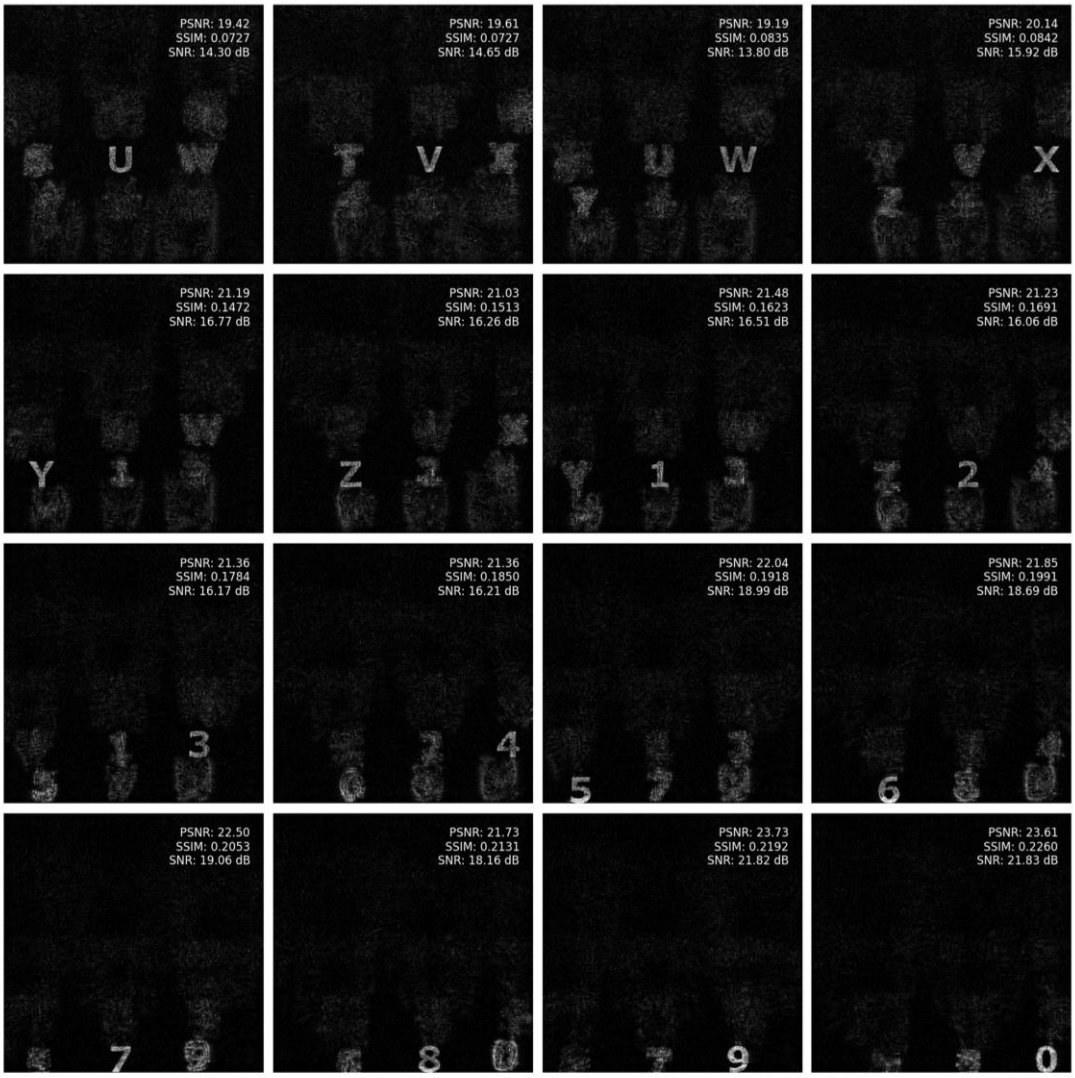
PSNR: 19.42
SSIM: 0.0727
SNR: 14.30 dB
PSNR: 19.61
SSIM: 0.0727
SNR: 14.65 dB
PSNR: 19.19
SSIM: 0.0835
SNR: 13.80 dB
PSNR: 20.14
SSIM: 0.0842
SNR: 15.92 dB
PSNR: 21.19
SSIM: 0.1472
SNR: 16.77 dB
PSNR: 21.03
SSIM: 0.1513
SNR: 16.26 dB
PSNR: 21.48
SSIM: 0.1623
SNR: 16.51 dB
PSNR: 21.23
SSIM: 0.1691
SNR: 16.06 dB
PSNR: 21.36
SSIM: 0.1784
SNR: 16.17 dB
PSNR: 21.36
SSIM: 0.1850
SNR: 16.21 dB
PSNR: 22.04
SSIM: 0.1918
SNR: 18.99 dB
PSNR: 21.85
SSIM: 0.1991
SNR: 18.69 dB
PSNR: 22.50
SSIM: 0.2053
SNR: 19.06 dB
PSNR: 21.73
SSIM: 0.2131
SNR: 18.16 dB
PSNR: 23.73
SSIM: 0.2192
SNR: 21.82 dB
PSNR: 23.61
SSIM: 0.2260
SNR: 21.83 dB

(b)

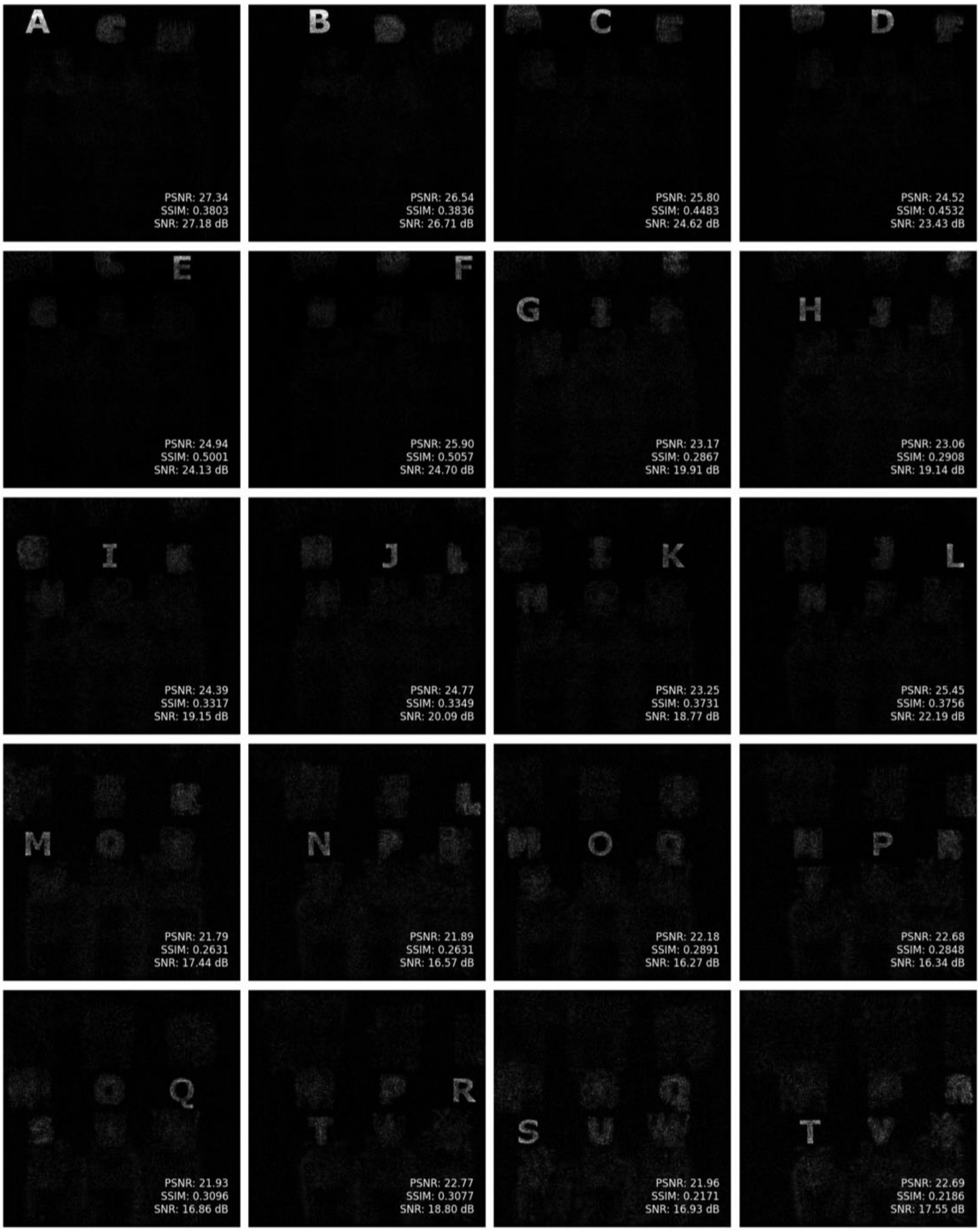
A
PSNR: 27.34
SSIM: 0.3803
SNR: 27.18 dB
B
PSNR: 26.54
SSIM: 0.3836
SNR: 26.71 dB
C
PSNR: 25.80
SSIM: 0.4483
SNR: 24.62 dB
D
PSNR: 24.52
SSIM: 0.4532
SNR: 23.43 dB
E
PSNR: 24.94
SSIM: 0.5001
SNR: 24.13 dB
F
PSNR: 25.90
SSIM: 0.5057
SNR: 24.70 dB
G
PSNR: 23.17
SSIM: 0.2867
SNR: 19.91 dB
H
PSNR: 23.06
SSIM: 0.2908
SNR: 19.14 dB
I
PSNR: 24.39
SSIM: 0.3317
SNR: 19.15 dB
J
PSNR: 24.77
SSIM: 0.3349
SNR: 20.09 dB
K
PSNR: 23.25
SSIM: 0.3731
SNR: 18.77 dB
L
PSNR: 25.45
SSIM: 0.3756
SNR: 22.19 dB
M
PSNR: 21.79
SSIM: 0.2631
SNR: 17.44 dB
N
PSNR: 21.89
SSIM: 0.2631
SNR: 16.57 dB
O
PSNR: 22.18
SSIM: 0.2891
SNR: 16.27 dB
P
PSNR: 22.68
SSIM: 0.2848
SNR: 16.34 dB
Q
PSNR: 21.93
SSIM: 0.3096
SNR: 16.86 dB
R
PSNR: 22.77
SSIM: 0.3077
SNR: 18.80 dB
S
PSNR: 21.96
SSIM: 0.2171
SNR: 16.93 dB
T
PSNR: 22.69
SSIM: 0.2186
SNR: 17.55 dB

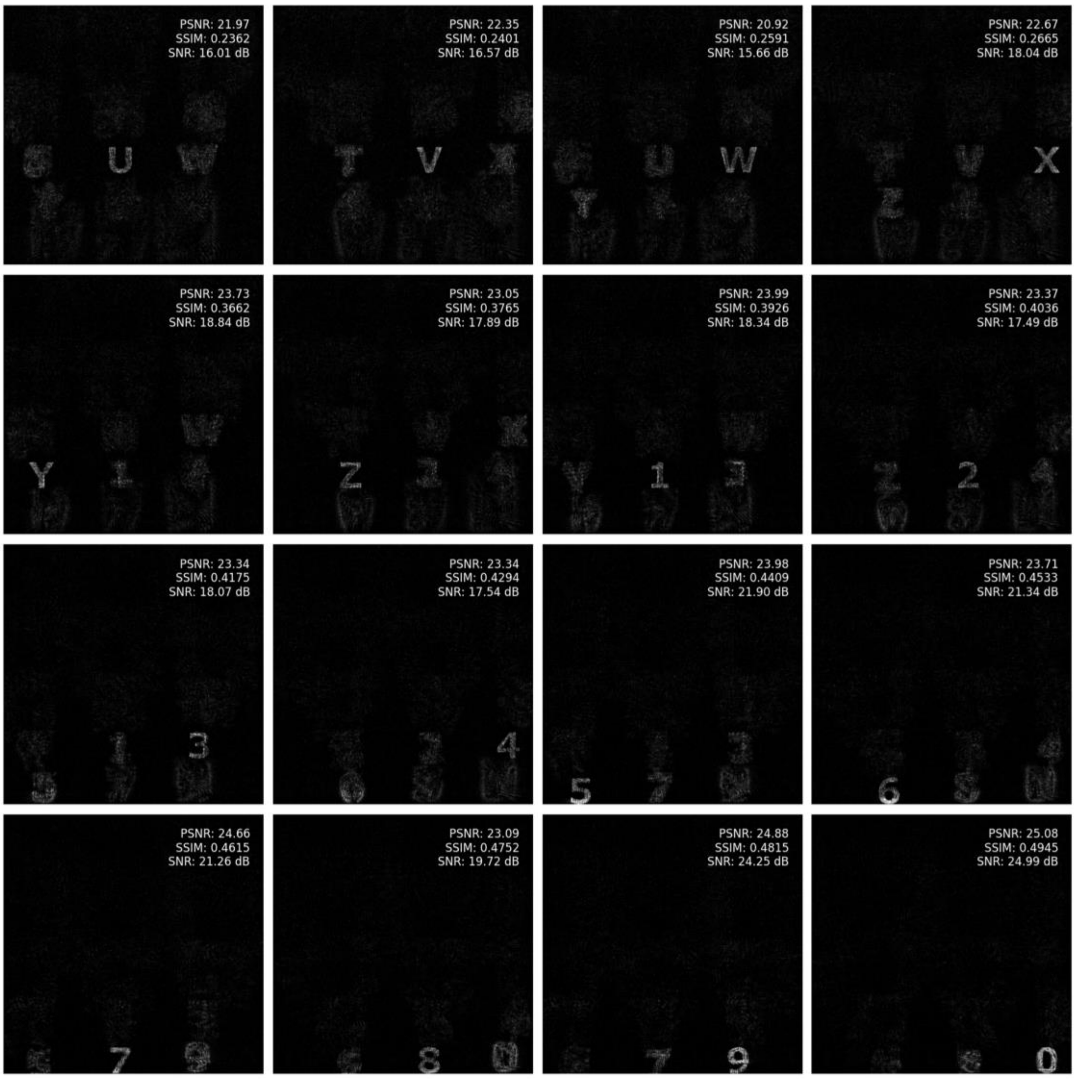

Figure S1. Analysis of PSNR, SSIM, and SNR for hologram simulations when incorporating noise suppresion loss:(a) Hologram image using only the traditional MSE loss without incorporating noise loss. (b) Hologram image using only the $\mathcal{L}_{\text{noise}}$ for background noise reduction.

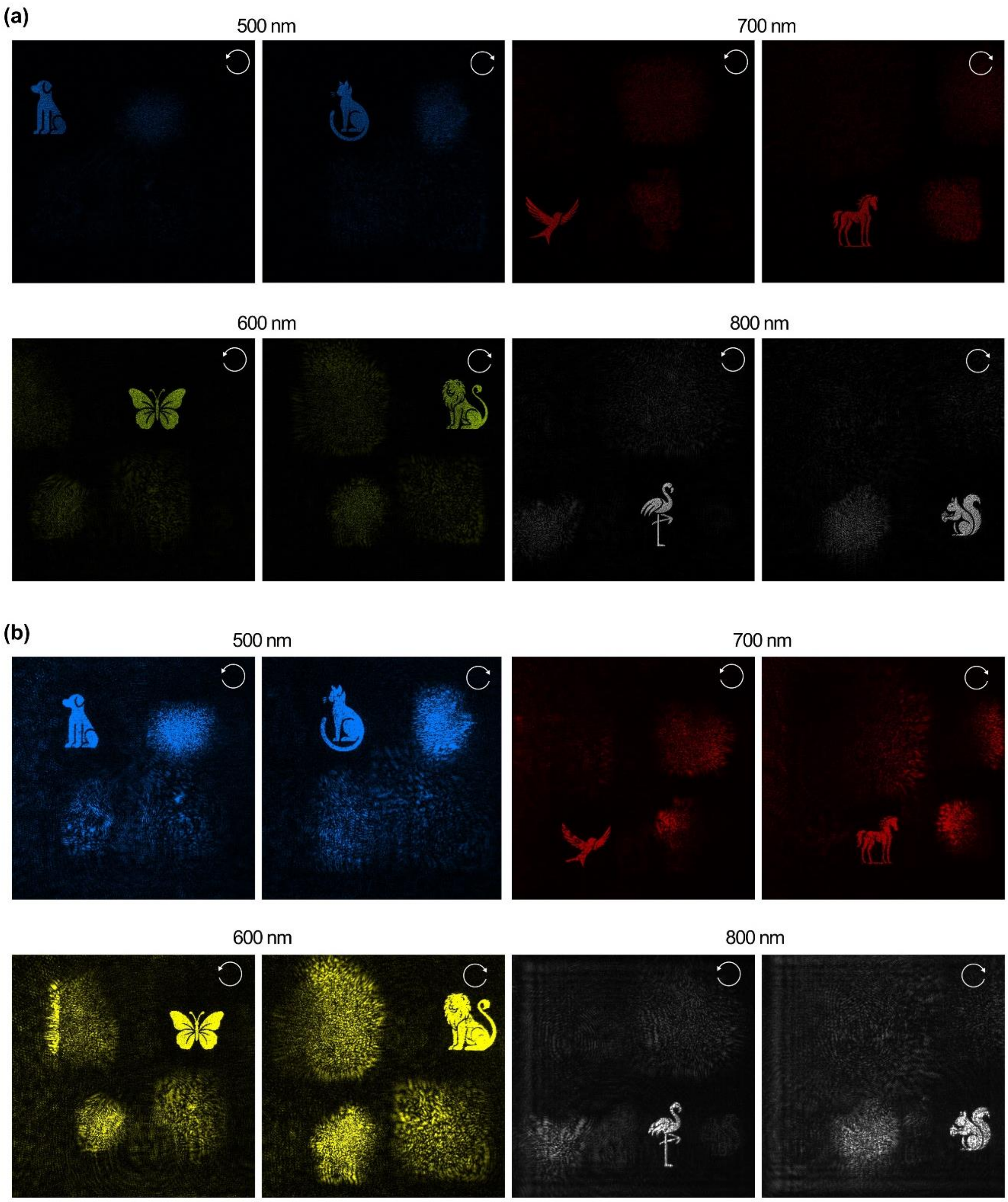

Figure S2. Full image of the 8-channel multiplexed hologram optimized using only MSE loss. (a) Simulated full image. (b) Experimental full image captured by sCMOS, showing the holographic output for both LCP and RCP incident light at 4 different wavelengths.

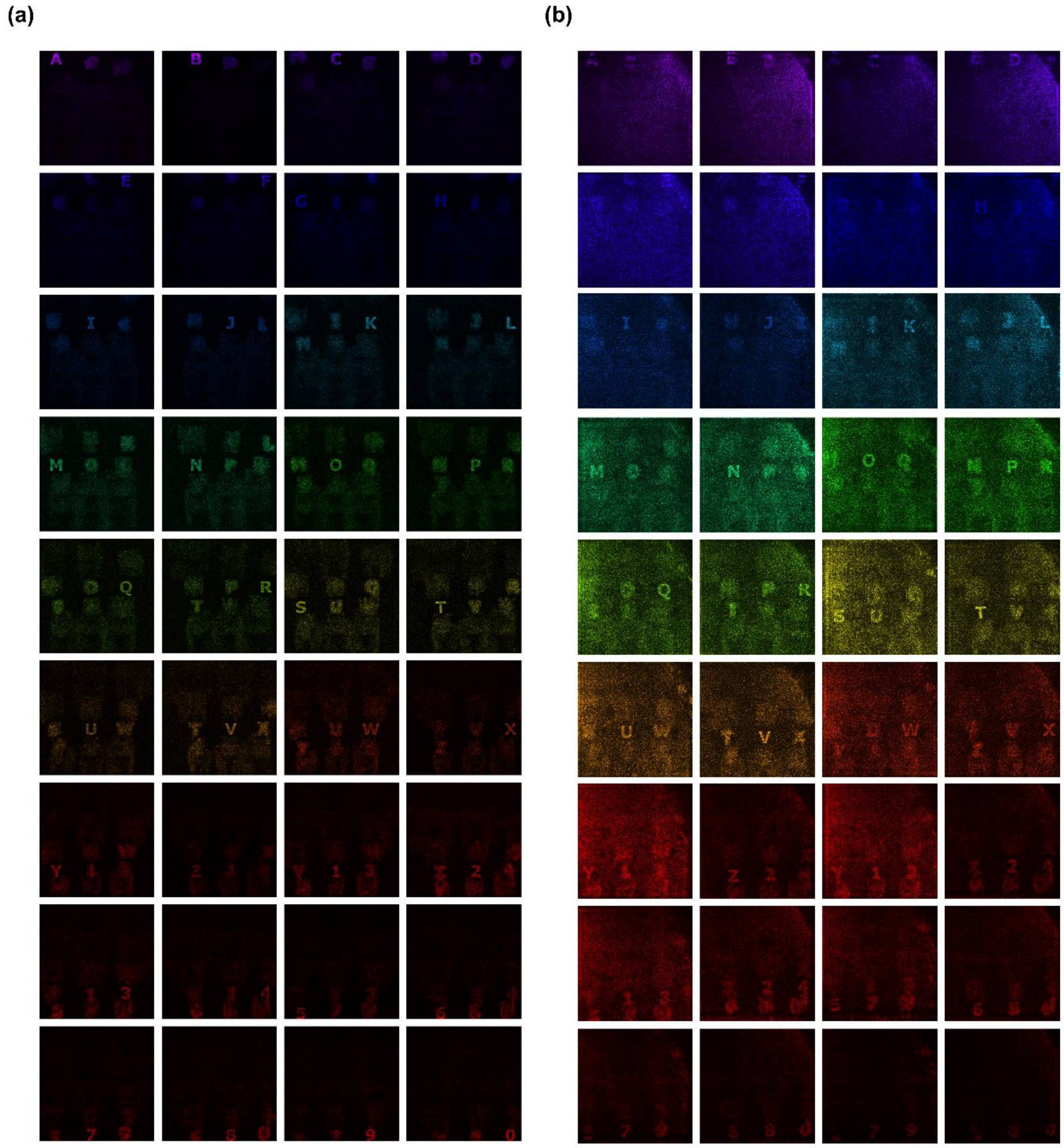

Figure S3. Full image of the 36-channel multiplexed hologram optimized using only MSE loss. (a) Simulated full image. (b) Experimental full image captured by sCMOS, showing the holographic output for both LCP and RCP incident light at 18 different wavelengths.

Figure S1(a) and **Figure S1(b)** present the results when only the MSE loss is implemented and when only the background noise loss term is considered, respectively. While incorporating the background noise loss term significantly reduces surrounding noise compared to using only the MSE loss, some crosstalk between adjacent channels remains visible. This issue was addressed by introducing an orthogonality loss term specifically calculated for adjacent wavelengths (Detailed images can be found in **Supplementary Note 14**).

The simulation and experimental results for the 8-channel hologram using only the MSE loss are presented in **Figure S2**, while the results for the 36-channel hologram using only the MSE loss are shown in **Figure S3**. These figures illustrate the impact of the loss functions on the holographic images. When comparing the contribution of each loss function, the results demonstrate that higher weight for λ_1, which corresponds to the MSE loss, ensures greater similarity within the ROI. However, this comes at the expense of reduced suppression of background noise. Conversely, increasing the weights of the noise-related losses, λ_2 and λ_3, effectively suppresses background noise and crosstalk between channels. However, overly emphasizing these noise-related losses may slightly compromise the clarity and fidelity of the ROI. To achieve optimal performance, careful balancing of the three weight parameters is necessary. For the final optimization, normalized weights were applied to the loss components, with λ_1 = 1, λ_2 = 0.5, and $\lambda_3 = 2.5 \times 10e^{-9}$. These values were chosen to achieve an optimal trade-off between image fidelity and noise suppression, ensuring high-quality reconstruction of the holographic images (Detailed images can be found in **Supplementary Note 9, Supplementary Note 13**).

Supporting Note 2. Rayleigh–Sommerfeld diffraction

The propagation of wave fields in free space is described by the wave equation. For time-harmonic fields, this equation is reduced to the Helmholtz equation, which governs the propagation of scalar wave fields in isotropic and homogeneous media. The Helmholtz equation is expressed as

$$\nabla^2 U(x,y,z) + k^2 U(x,y,z) = 0 \tag{1}$$

where $U(x,y,z)$ represents the scalar wave field, $k = 2\pi/\lambda$ is the wave number, and λ is the wavelength.

One approach to solving the Helmholtz equation is to express the wave field in terms of spherical wavefronts. This method is based on the Huygens–Fresnel principle, in which each point on a wavefront is considered as a source of secondary spherical wavelets. The Rayleigh–Sommerfeld diffraction integral uses Green's function and boundary conditions to express how these wavelets interfere to form the propagated wavefront in the observation plane. The diffraction integral combines the contributions of the spherical wavelets generated at every point on the input plane. The Rayleigh–Sommerfeld diffraction formula is expressed as follows:

$$U(x,y,z) = \frac{1}{2\pi}\int_{-\infty}^{\infty}\int_{-\infty}^{\infty} U(x_0,y_0,0)\left(\frac{1}{r} - jk\right)\frac{e^{jkr}}{r} z dx_0 dy_0 \tag{2}$$

To efficiently compute the diffraction integral, the Fourier transform can be applied to simplify the process. The Fourier transform converts the complex spatial-domain integral into a pointwise multiplication in the frequency domain, which is computationally efficient. The results are then transformed back into the spatial domain using an inverse Fourier transform. This approach can be expressed as follows:

$$U(x,y,z) = \mathcal{F}^{-1}\{\mathcal{F}[U(x_0,y_0,0)] \cdot \mathcal{F}[h(x,y,z)]\} \tag{3}$$

In the frequency domain, the wave-field propagation can be described using the optical transfer function, which represents how the wave propagates through a given medium. The inverse Fourier transform of the optical transfer function provides the impulse response of the system, which describes how the wavefront of a point source propagates through space:

$$h(x,y,z) = \frac{1}{2\pi}\frac{\exp(jkr)}{r}\frac{z}{r}\left(\frac{1}{r} - jk\right)$$

The convolution in the spatial domain is converted into simple multiplication in the frequency domain. This method significantly improves the computational efficiency, especially for large-scale diffraction problems.

Supporting Note 3. Gradient-based iterative optimization results

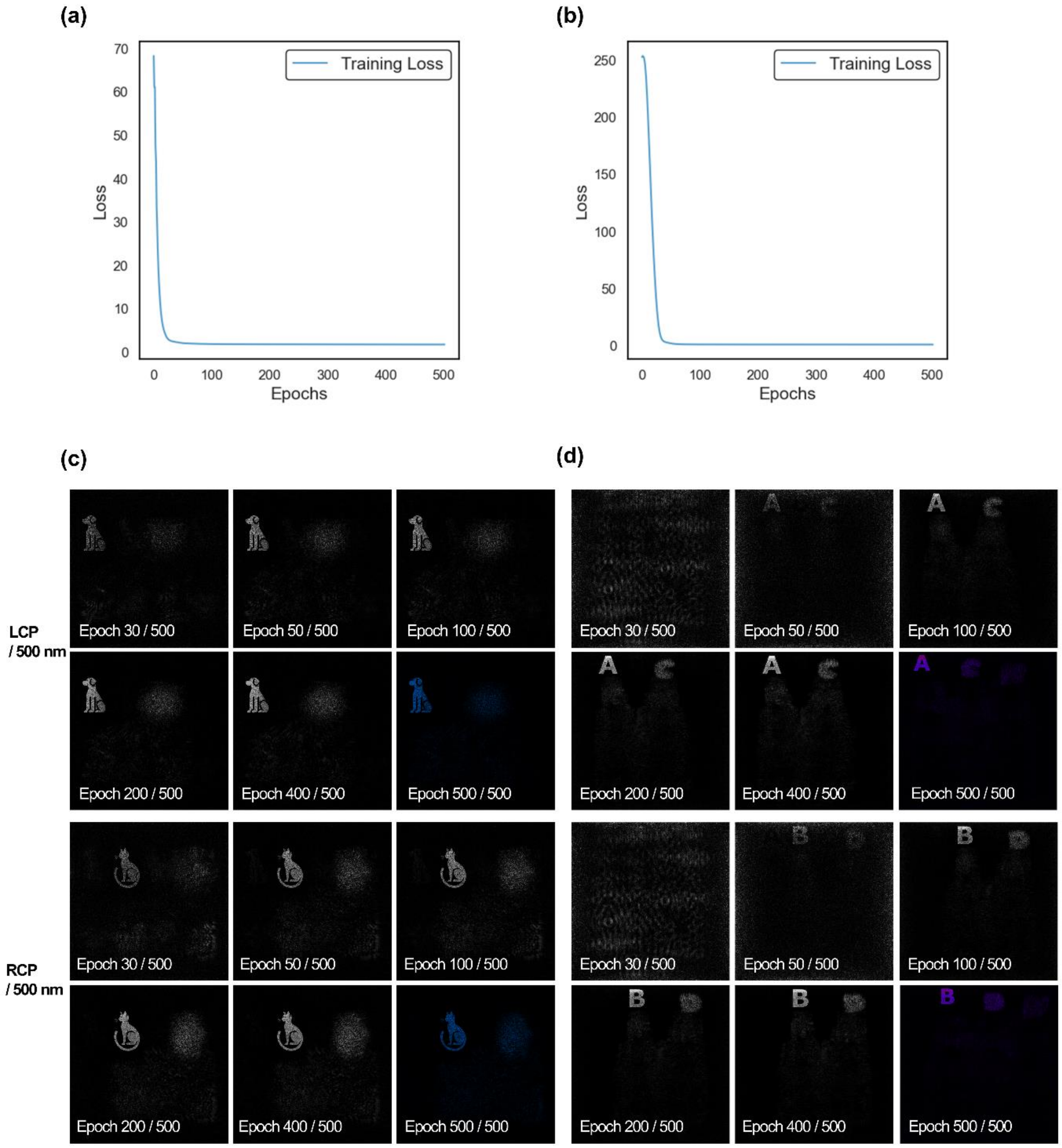

Figure S4. Training loss and image comparisons across epochs for multiplexed holograms. (a) Training loss (c) and epoch-wise image comparisons for the 8-channel multiplexed hologram (animal images). (b) Training loss (d) and epoch-wise image comparisons for the 36-channel multiplexed hologram (alphabet images).

Supporting Note 4. Optical properties of SiN_x used for simulation.

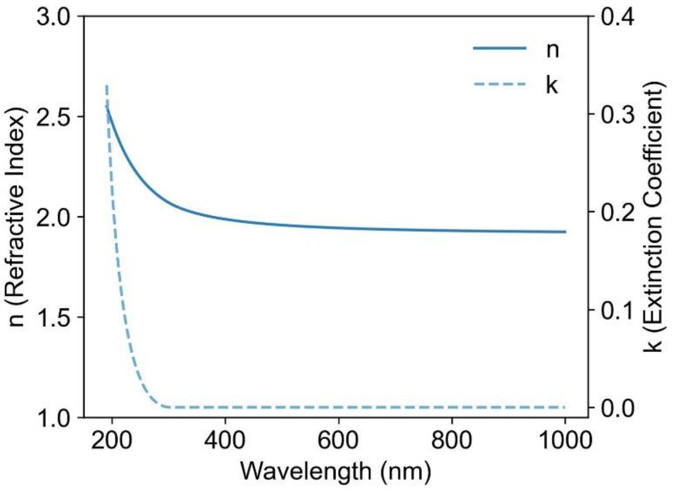

Figure S5. Ellipsometry data of measured refractive index and extinction coefficient. SiN_x shows transparency due to low k in the broadband wavelength range, from the visible to NIR region.

Supporting Note 5. Simulation results of conversion efficiency map

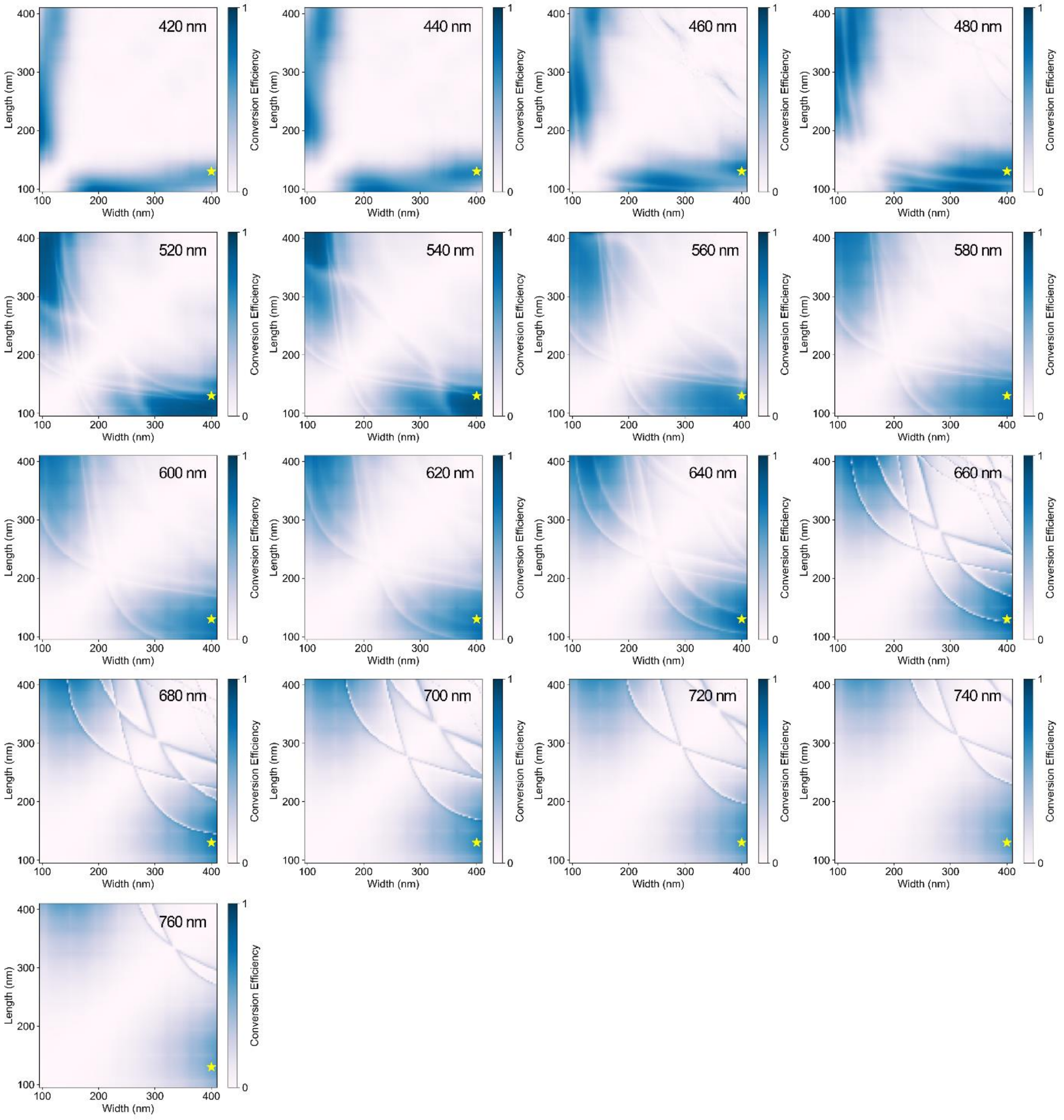

Figure S6. Conversion efficiency map for wavelengths λ = 420, 440, 460, 480, 520, 540, 560, 580, 600, 620, 640, 660, 680, 700, 720, 740, and 760 nm, calculated using rigorous coupled-wave analysis at a fixed height of 950 nm and period of 450 nm for various widths and lengths.

Supporting Note 6. Simulation results of E-field distribution

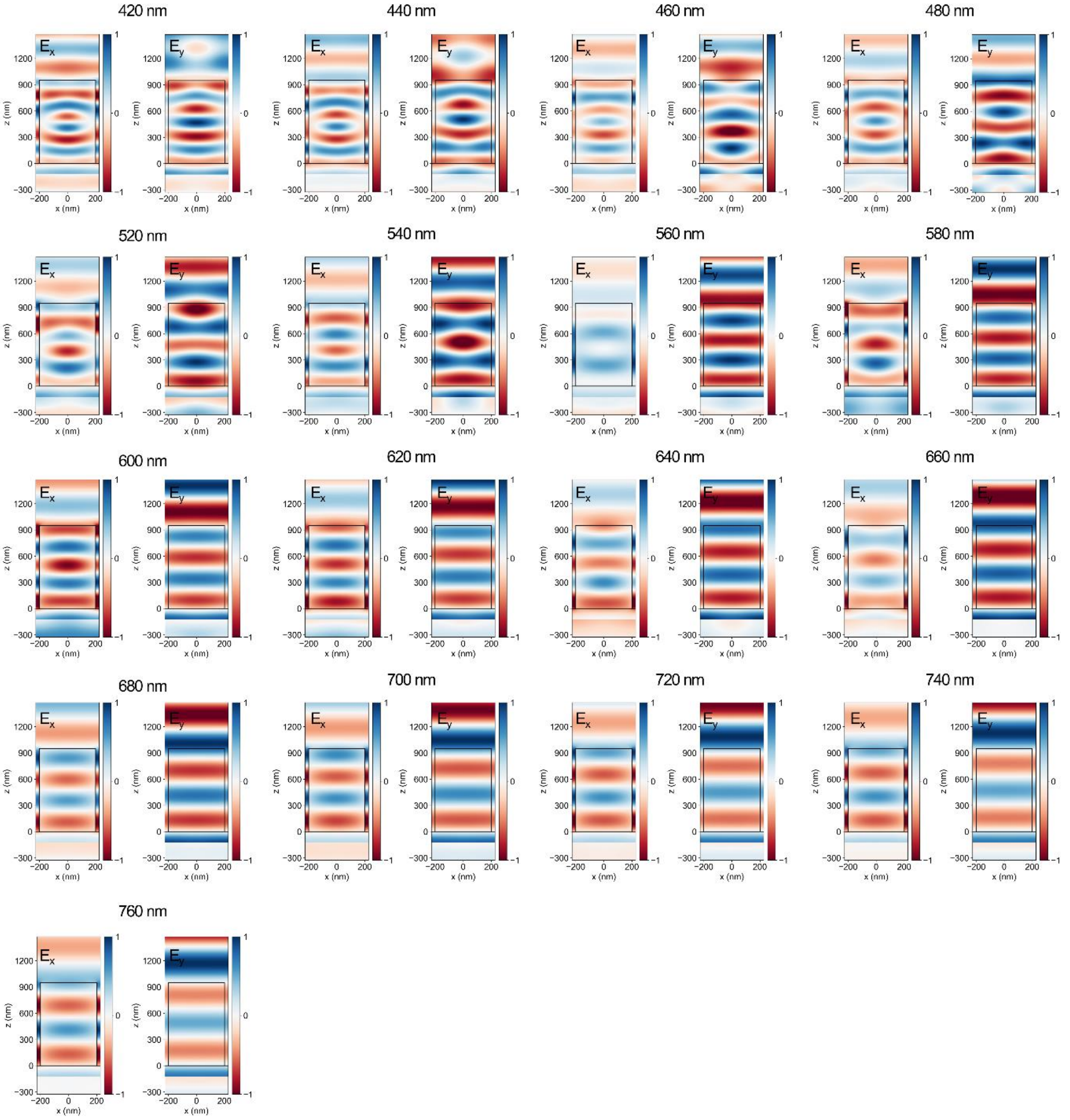

Figure S7. Electric-field distribution in the yz plane using finite-difference time-domain simulations for wavelengths λ = 420, 440, 460, 480, 520, 540, 560, 580, 600, 620, 640, 660, 680, 700, 720, 740, and 760 nm, with results shown for both x- and y-polarized incident light. The structure dimensions used in the calculations have a width of 400 nm, length of 130 nm, height of 950 nm, and period of 450 nm.

Supporting Note 7. Fabrication procedure

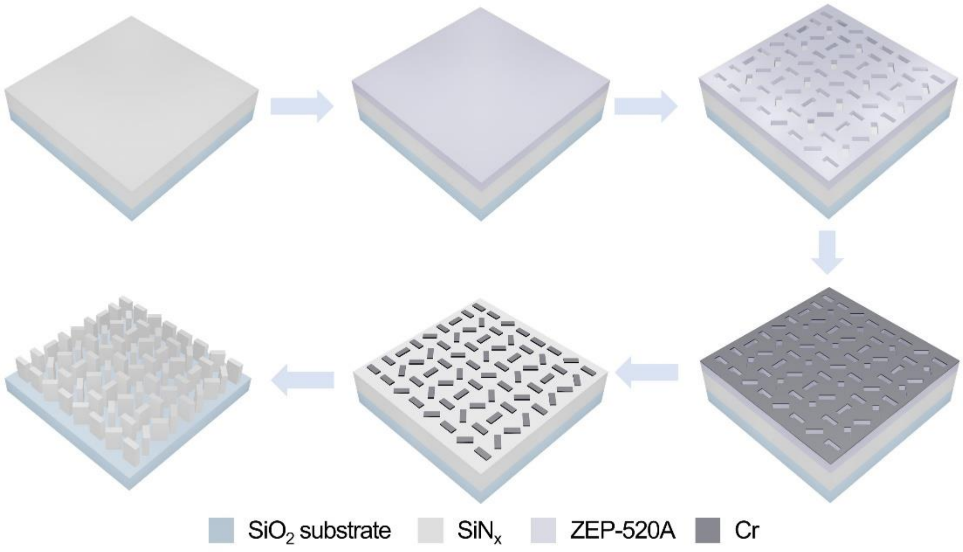

Figure S8. Fabrication procedure of the designed metasurface.

Supporting Note 8. Fabricated metasurface image

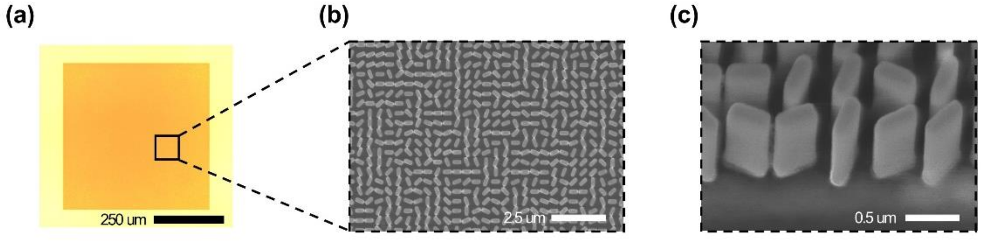

Figure S9. Fabricated 36-channel multiplexed hologram. (a) Optical-microscopy image of the entire metasurface at ×20 magnification, (b) top-view SEM image at ×10k, and (c) tilted view of SEM image at ×50k.

Supporting Note 9. Simulated and experimental full image of 8-channel multiplexing hologram

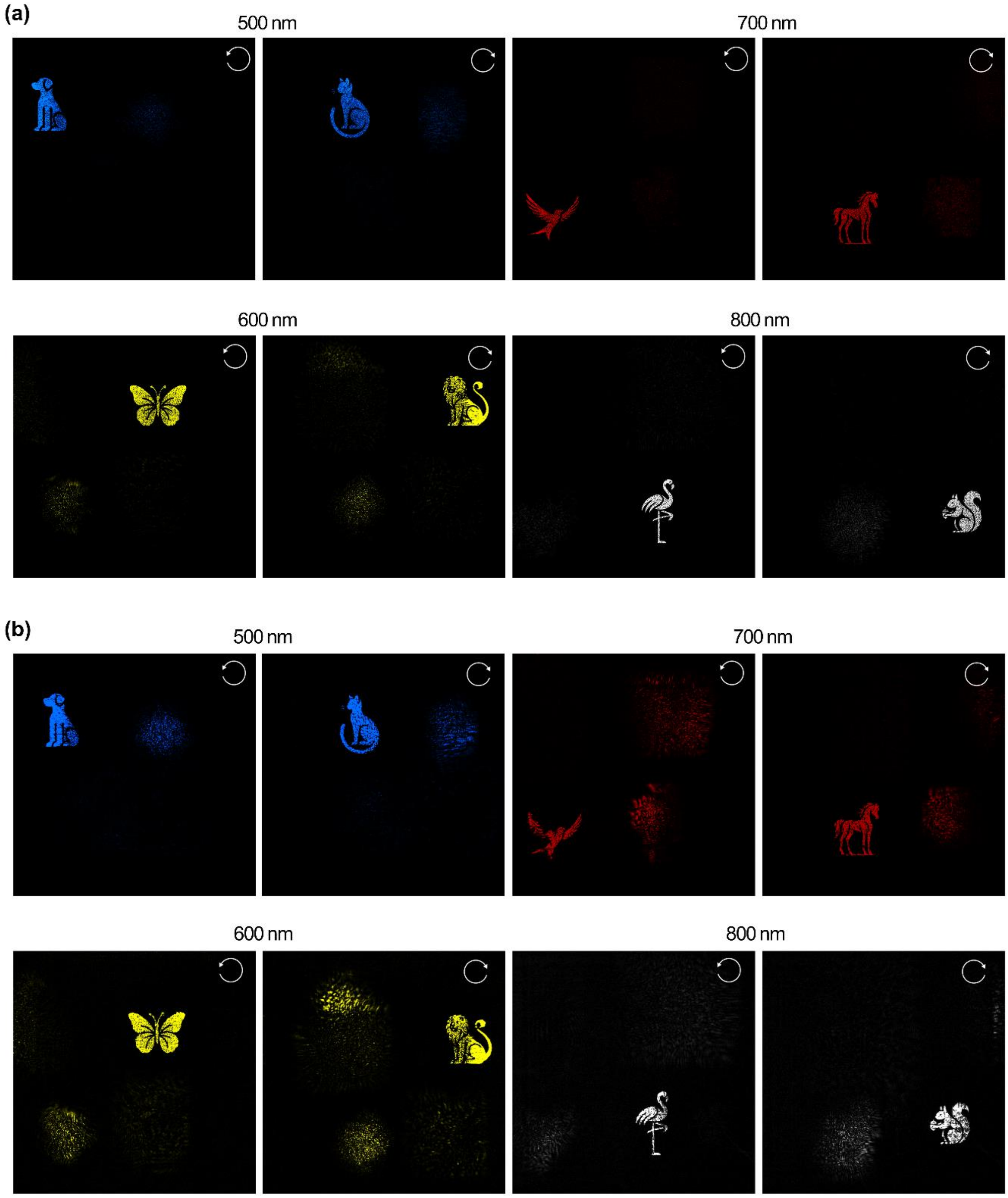

Figure S10. Full image of the 8-channel multiplexed hologram fully optimized designs, incorporating both noise-related loss terms, (a) Simulated full image. (b) Experimental full image captured by sCMOS, showing the holographic output for both LCP and RCP incident light at 4 different wavelengths.

Supporting Note 10. Comparison results of 8-channel multiplexed hologram between non-discretized and 8-level discretized phase simulations

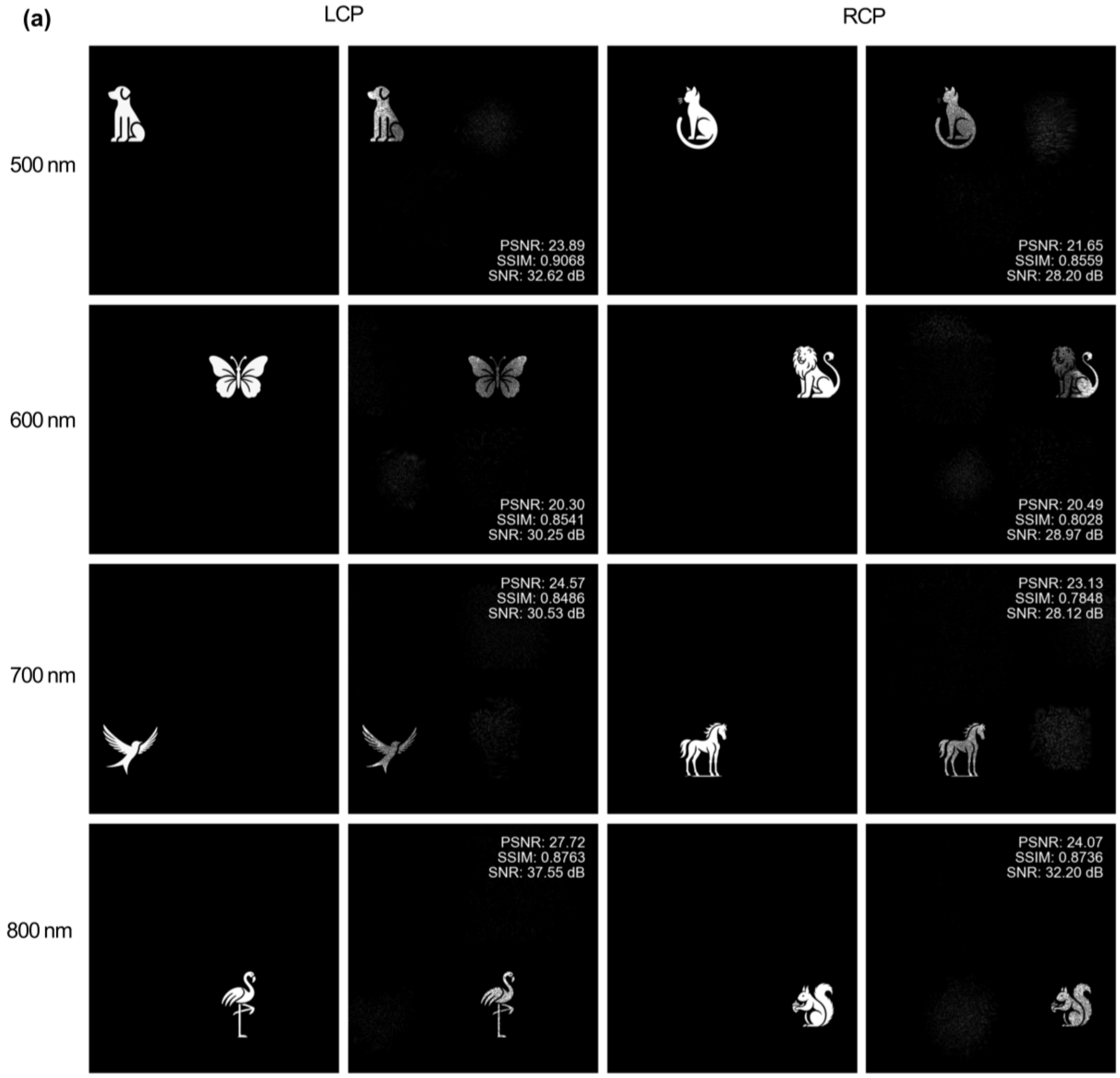

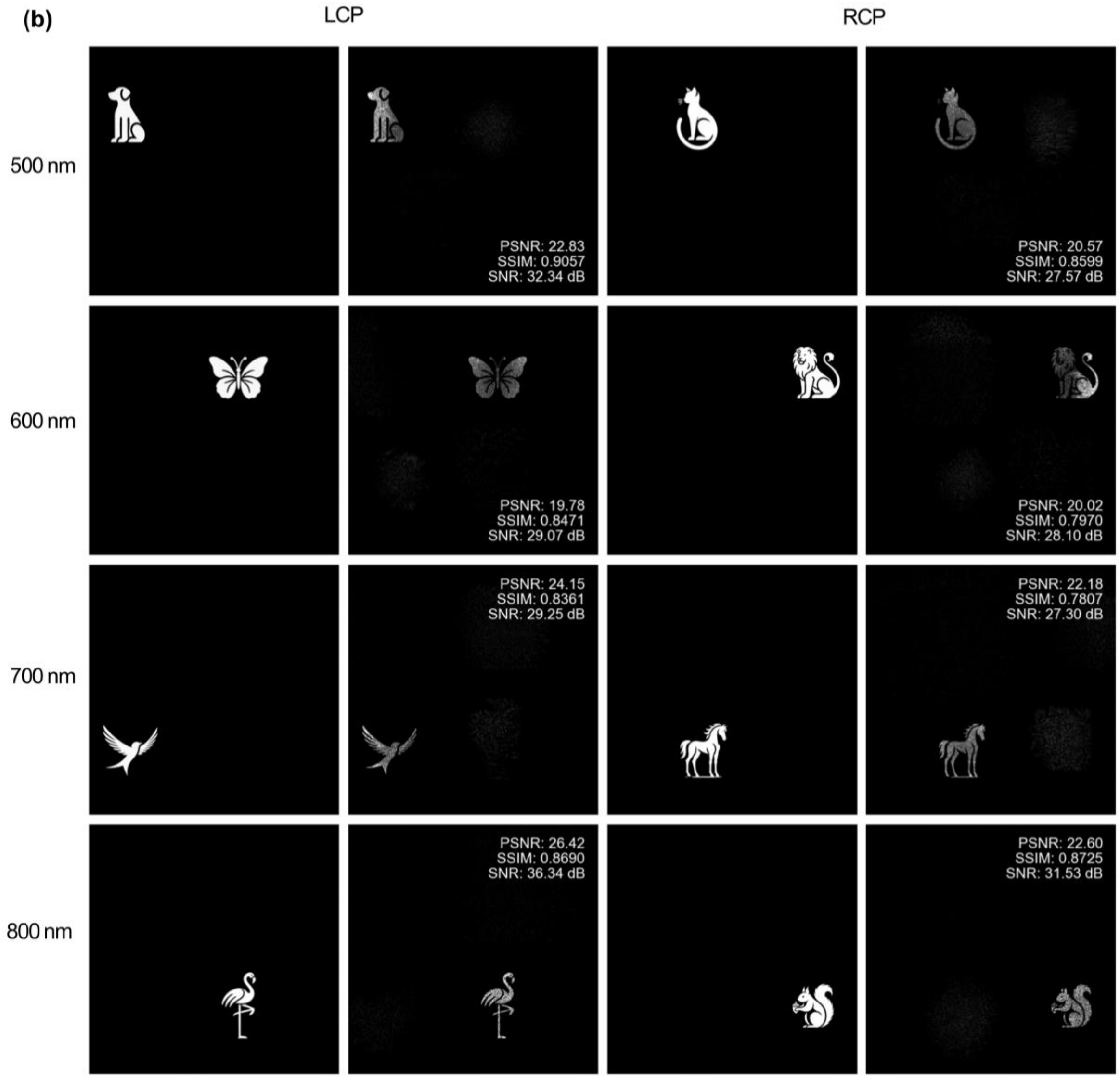

Figure S11. Comparison of PSNR, SSIM, and SNR values for the 8-channel multiplexed hologram (animal images) between (a) continuous (non-discretized) and (b) 8-level discretized phase simulations.

Supporting Note 11. Comparison results of 8-channel multiplexed hologram for specific region

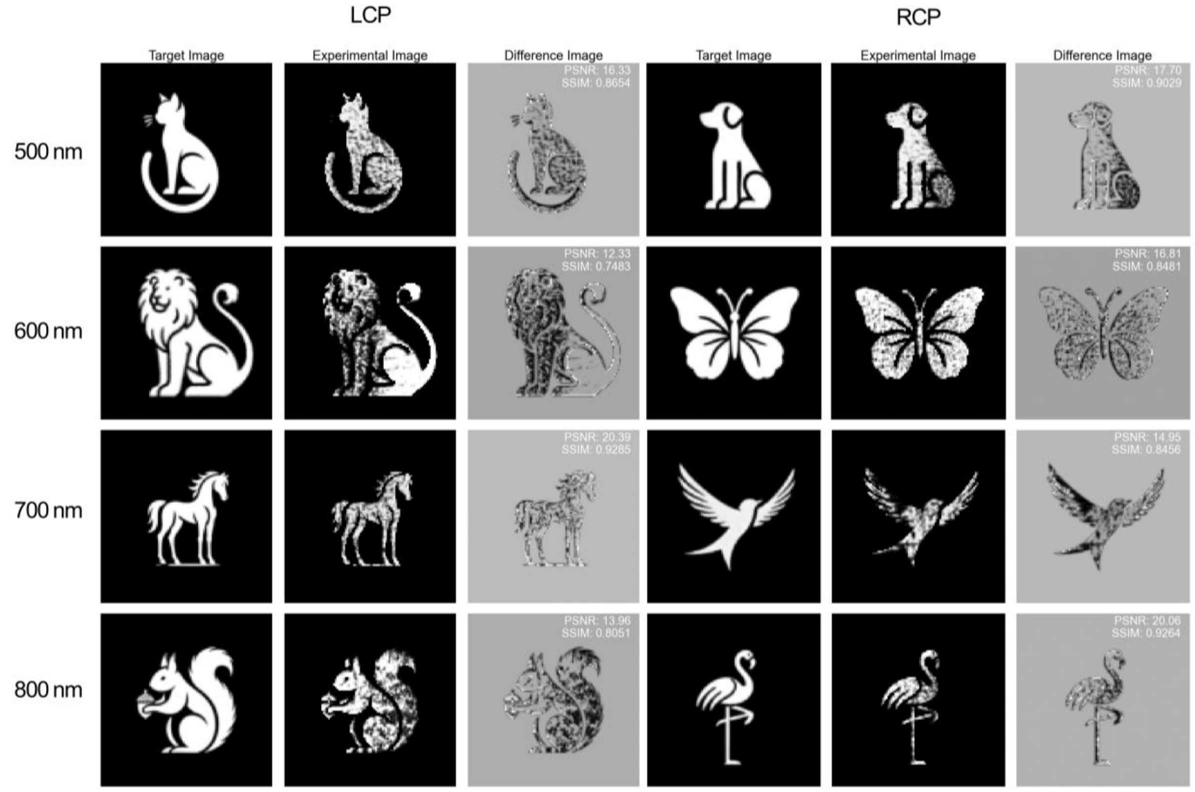

Figure S12. Comparison of PSNR and SSIM for the region of interest (here, animal image) in the 8-channel multiplexed hologram. Results are shown for both LCP and RCP incident light, comparing the target, experimental, and the difference images (the discrepancy between the two) at four different wavelengths (500, 600, 700, and 800 nm).

Supporting Note 12. Data analysis of full image from experimental results for the 8-channel hologram

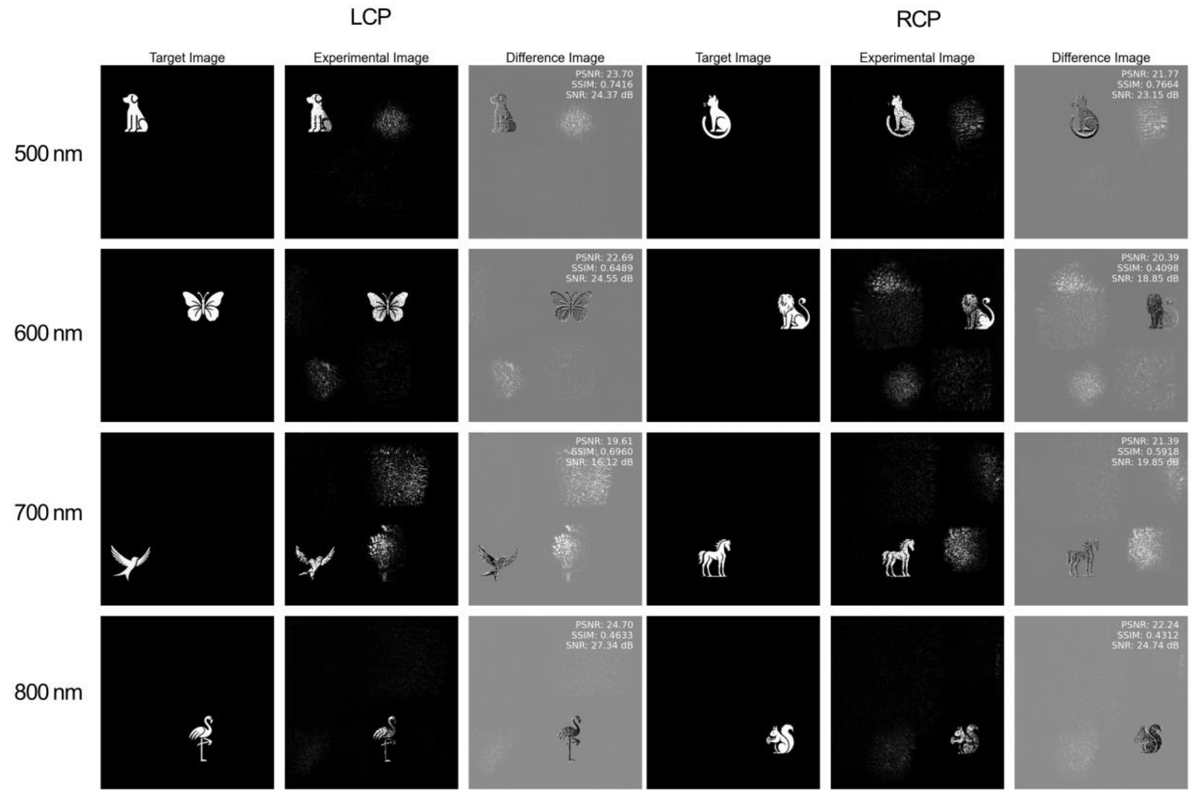

Figure S13. Comparison of PSNR, SSIM and SNR for full experimental image in the 8-channel multiplexed hologram. Results are shown for both LCP and RCP incident light, comparing the target, experimental, and the difference images (the discrepancy between the two) at four different wavelengths (500, 600, 700, and 800 nm).

Supporting Note 13. Simulated and experimental full image of 36-channel multiplexing hologram

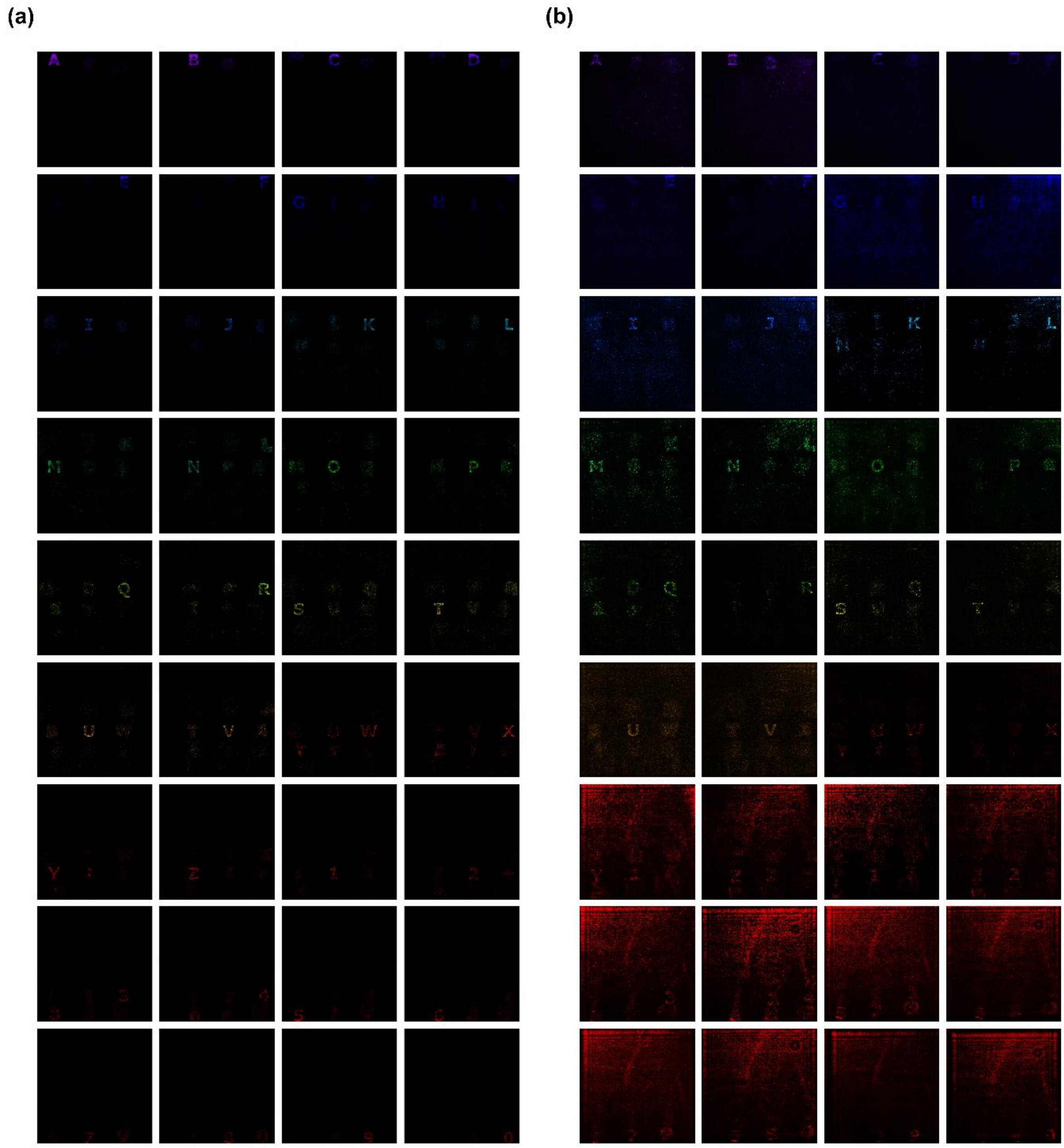

Figure S14. Full image of the 36-channel multiplexing hologram. (a) Simulated full image. (b) Experimental full image captured by CCD, showing the holographic output for both LCP and RCP incident light at 18 different wavelengths.

Supporting Note 14. PSNR, SSIM, and SNR values for the full 36 channel hologram images

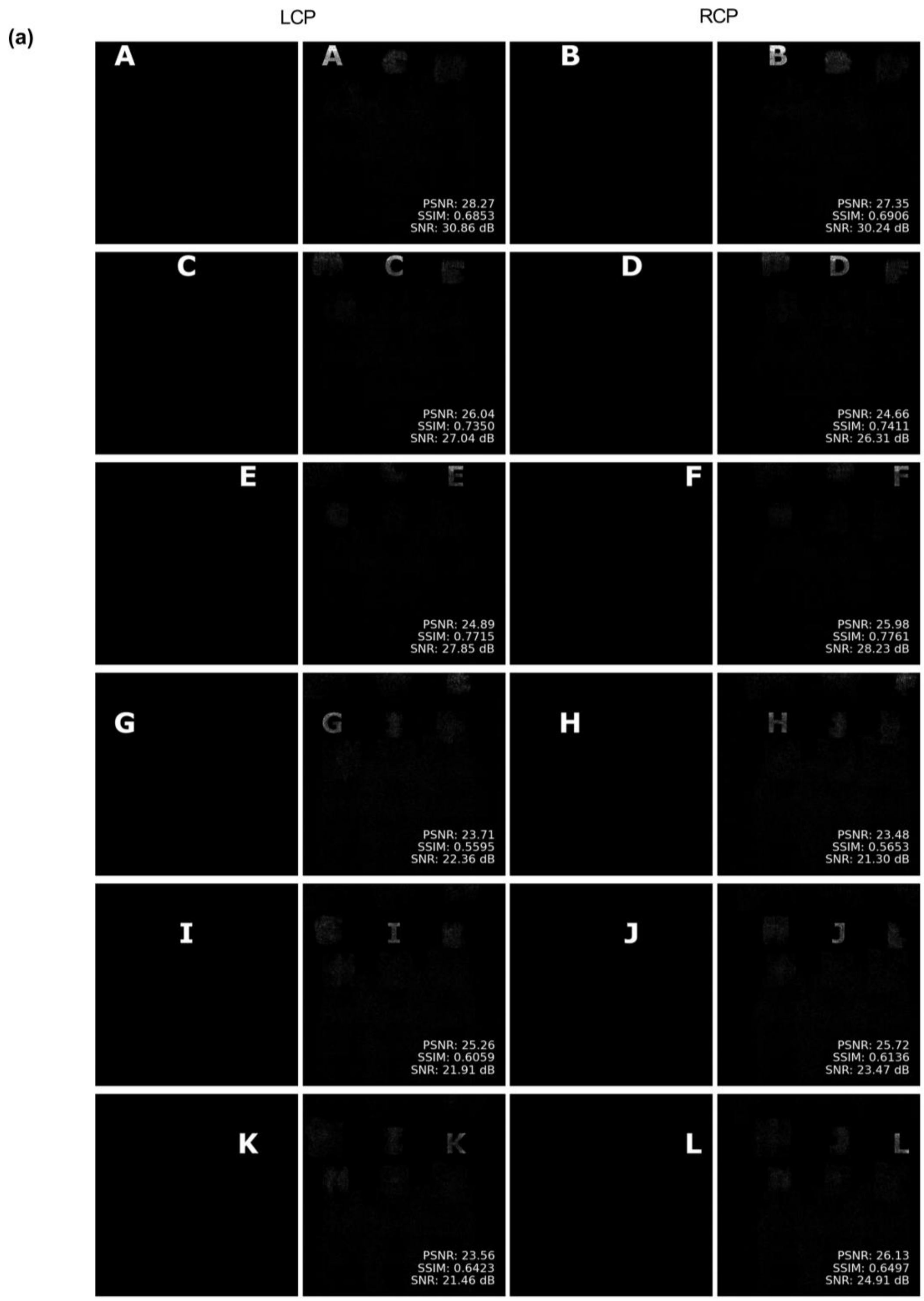

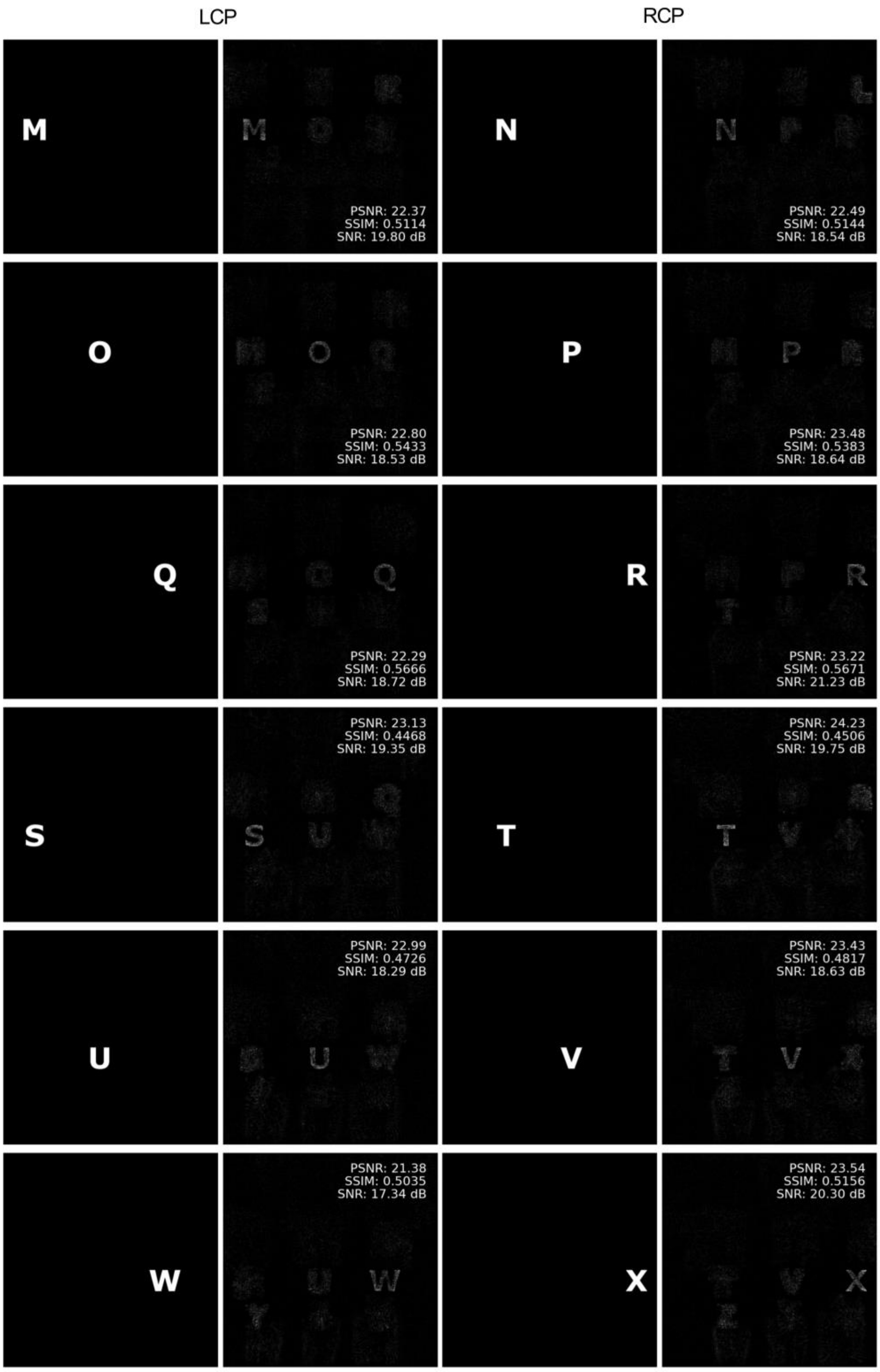
LCP
RCP
M
N
PSNR: 22.37
SSIM: 0.5114
SNR: 19.80 dB
PSNR: 22.49
SSIM: 0.5144
SNR: 18.54 dB
O
P
PSNR: 22.80
SSIM: 0.5433
SNR: 18.53 dB
PSNR: 23.48
SSIM: 0.5383
SNR: 18.64 dB
Q
R
PSNR: 22.29
SSIM: 0.5666
SNR: 18.72 dB
PSNR: 23.22
SSIM: 0.5671
SNR: 21.23 dB
S
T
PSNR: 23.13
SSIM: 0.4468
SNR: 19.35 dB
PSNR: 24.23
SSIM: 0.4506
SNR: 19.75 dB
U
V
PSNR: 22.99
SSIM: 0.4726
SNR: 18.29 dB
PSNR: 23.43
SSIM: 0.4817
SNR: 18.63 dB
W
X
PSNR: 21.38
SSIM: 0.5035
SNR: 17.34 dB
PSNR: 23.54
SSIM: 0.5156
SNR: 20.30 dB

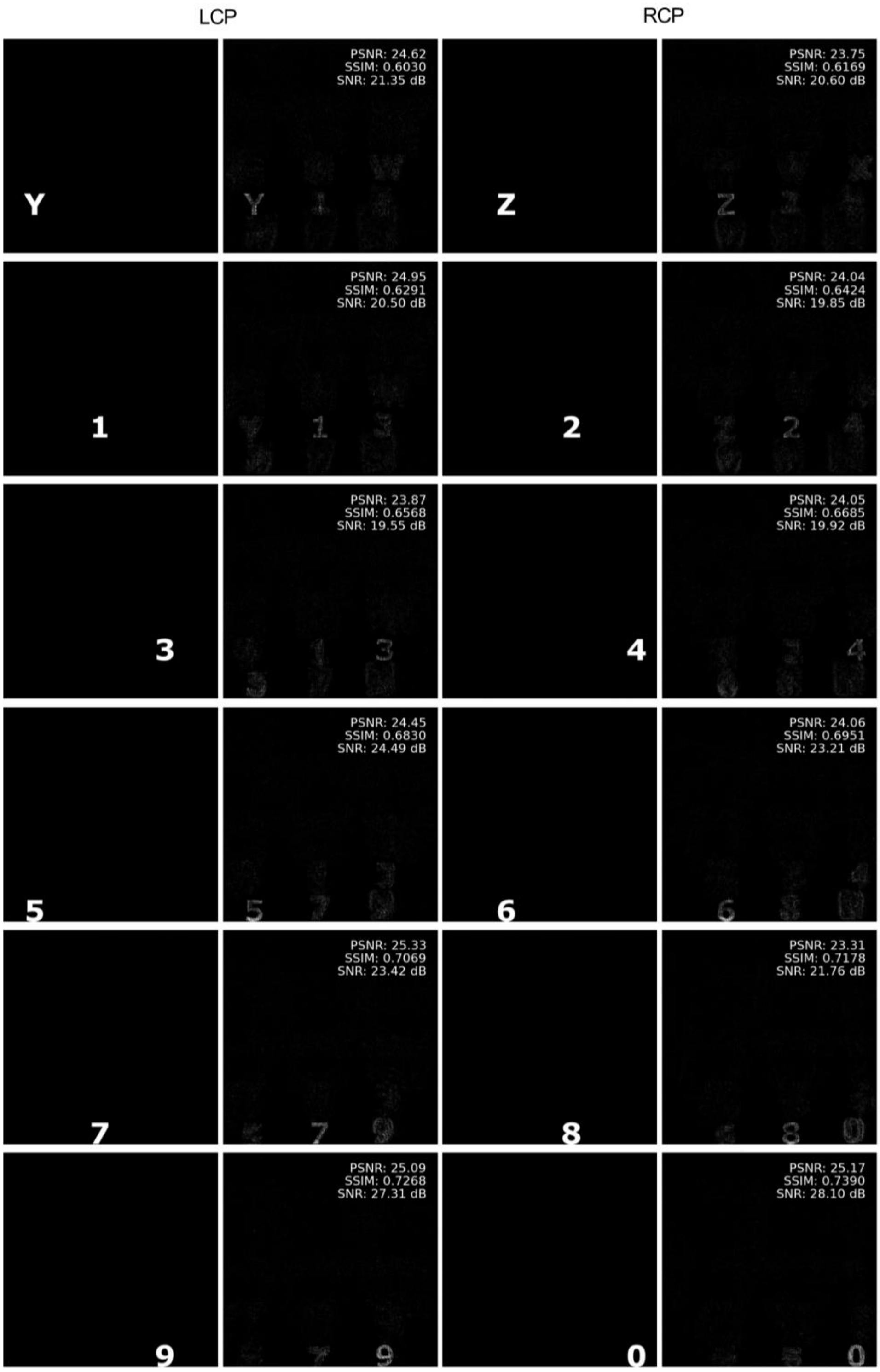
LCP
RCP
Y
PSNR: 24.62
SSIM: 0.6030
SNR: 21.35 dB
Z
PSNR: 23.75
SSIM: 0.6169
SNR: 20.60 dB
1
PSNR: 24.95
SSIM: 0.6291
SNR: 20.50 dB
2
PSNR: 24.04
SSIM: 0.6424
SNR: 19.85 dB
3
PSNR: 23.87
SSIM: 0.6568
SNR: 19.55 dB
4
PSNR: 24.05
SSIM: 0.6685
SNR: 19.92 dB
5
PSNR: 24.45
SSIM: 0.6830
SNR: 24.49 dB
6
PSNR: 24.06
SSIM: 0.6951
SNR: 23.21 dB
7
PSNR: 25.33
SSIM: 0.7069
SNR: 23.42 dB
8
PSNR: 23.31
SSIM: 0.7178
SNR: 21.76 dB
9
PSNR: 25.09
SSIM: 0.7268
SNR: 27.31 dB
0
PSNR: 25.17
SSIM: 0.7390
SNR: 28.10 dB

(b)

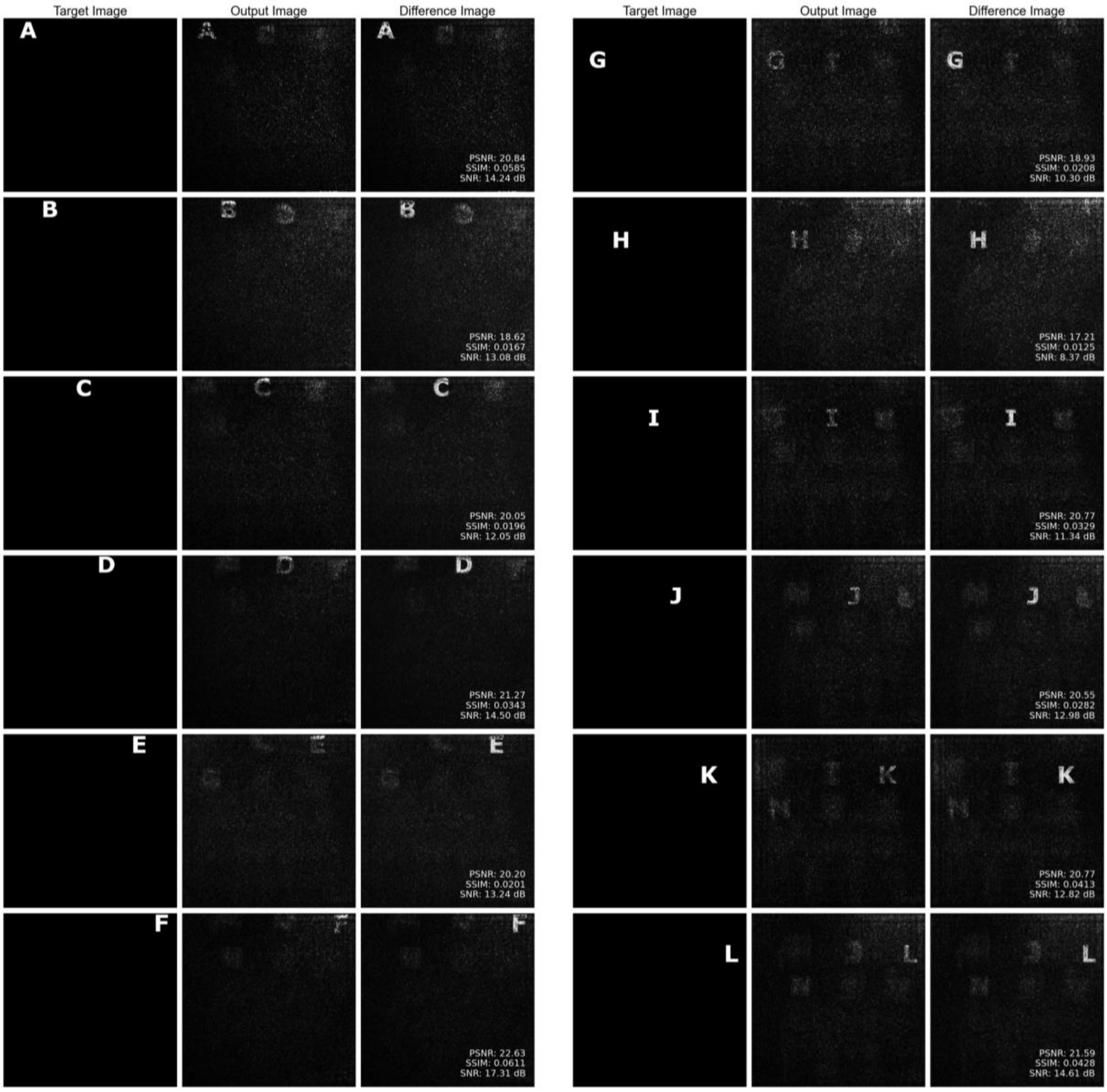
Target Image
Output Image
Difference Image
Target Image
Output Image
Difference Image
A
PSNR: 20.84
SSIM: 0.0585
SNR: 14.24 dB
G
PSNR: 18.93
SSIM: 0.0208
SNR: 10.30 dB
B
PSNR: 18.62
SSIM: 0.0167
SNR: 13.08 dB
H
PSNR: 17.21
SSIM: 0.0125
SNR: 8.37 dB
C
PSNR: 20.05
SSIM: 0.0196
SNR: 12.05 dB
I
PSNR: 20.77
SSIM: 0.0329
SNR: 11.34 dB
D
PSNR: 21.27
SSIM: 0.0343
SNR: 14.50 dB
J
PSNR: 20.55
SSIM: 0.0282
SNR: 12.98 dB
E
PSNR: 20.20
SSIM: 0.0201
SNR: 13.24 dB
K
PSNR: 20.77
SSIM: 0.0413
SNR: 12.82 dB
F
PSNR: 22.63
SSIM: 0.0611
SNR: 17.31 dB
L
PSNR: 21.59
SSIM: 0.0428
SNR: 14.61 dB

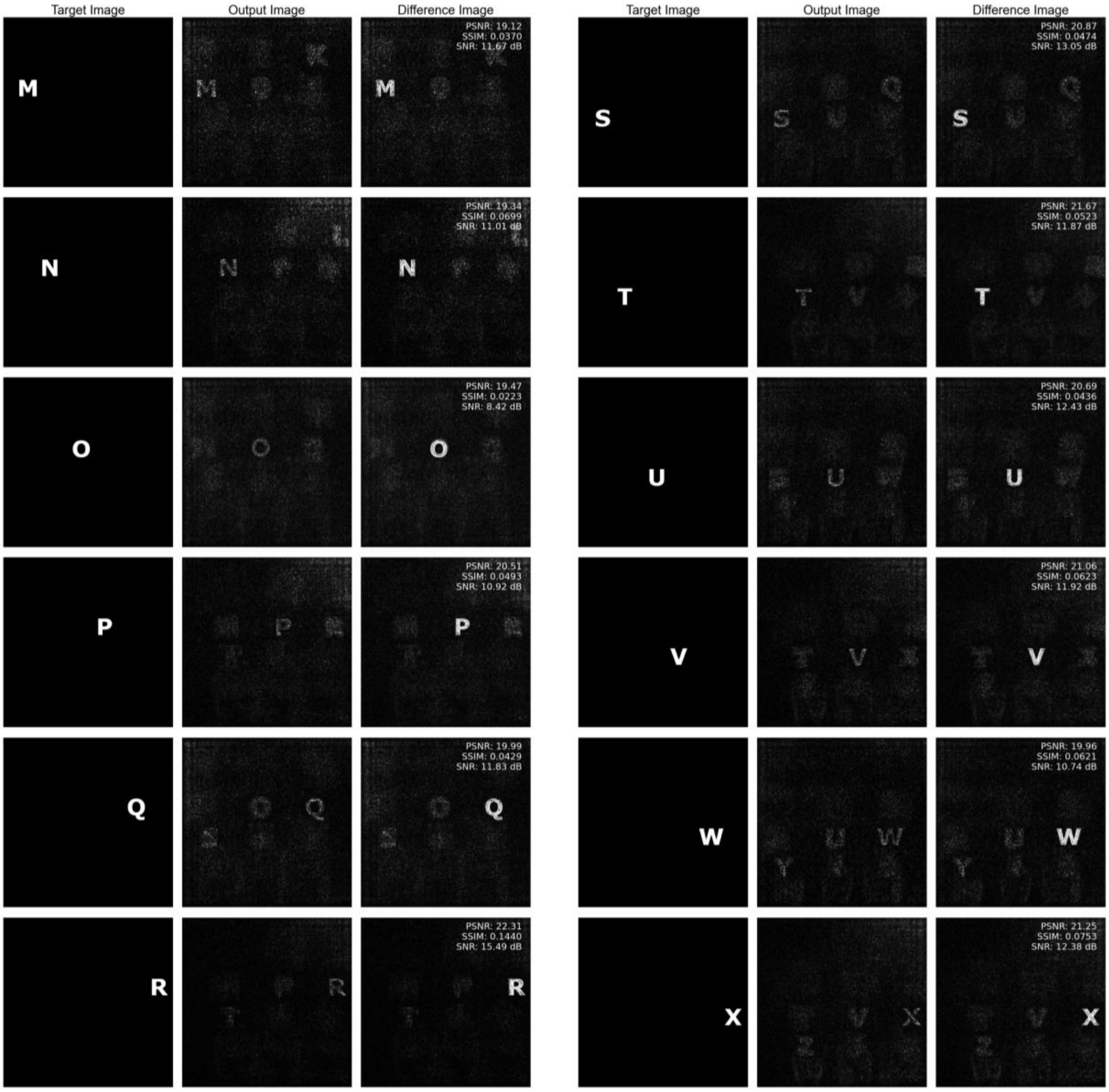
Target Image
Output Image
Difference Image
PSNR: 19.12
SSIM: 0.0370
SNR: 11.67 dB
PSNR: 19.34
SSIM: 0.0699
SNR: 11.01 dB
PSNR: 19.47
SSIM: 0.0223
SNR: 8.42 dB
PSNR: 20.51
SSIM: 0.0493
SNR: 10.92 dB
PSNR: 19.99
SSIM: 0.0429
SNR: 11.83 dB
PSNR: 22.31
SSIM: 0.1440
SNR: 15.49 dB
PSNR: 20.87
SSIM: 0.0474
SNR: 13.05 dB
PSNR: 21.67
SSIM: 0.0523
SNR: 11.87 dB
PSNR: 20.69
SSIM: 0.0436
SNR: 12.43 dB
PSNR: 21.06
SSIM: 0.0623
SNR: 11.92 dB
PSNR: 19.96
SSIM: 0.0621
SNR: 10.74 dB
PSNR: 21.25
SSIM: 0.0753
SNR: 12.38 dB

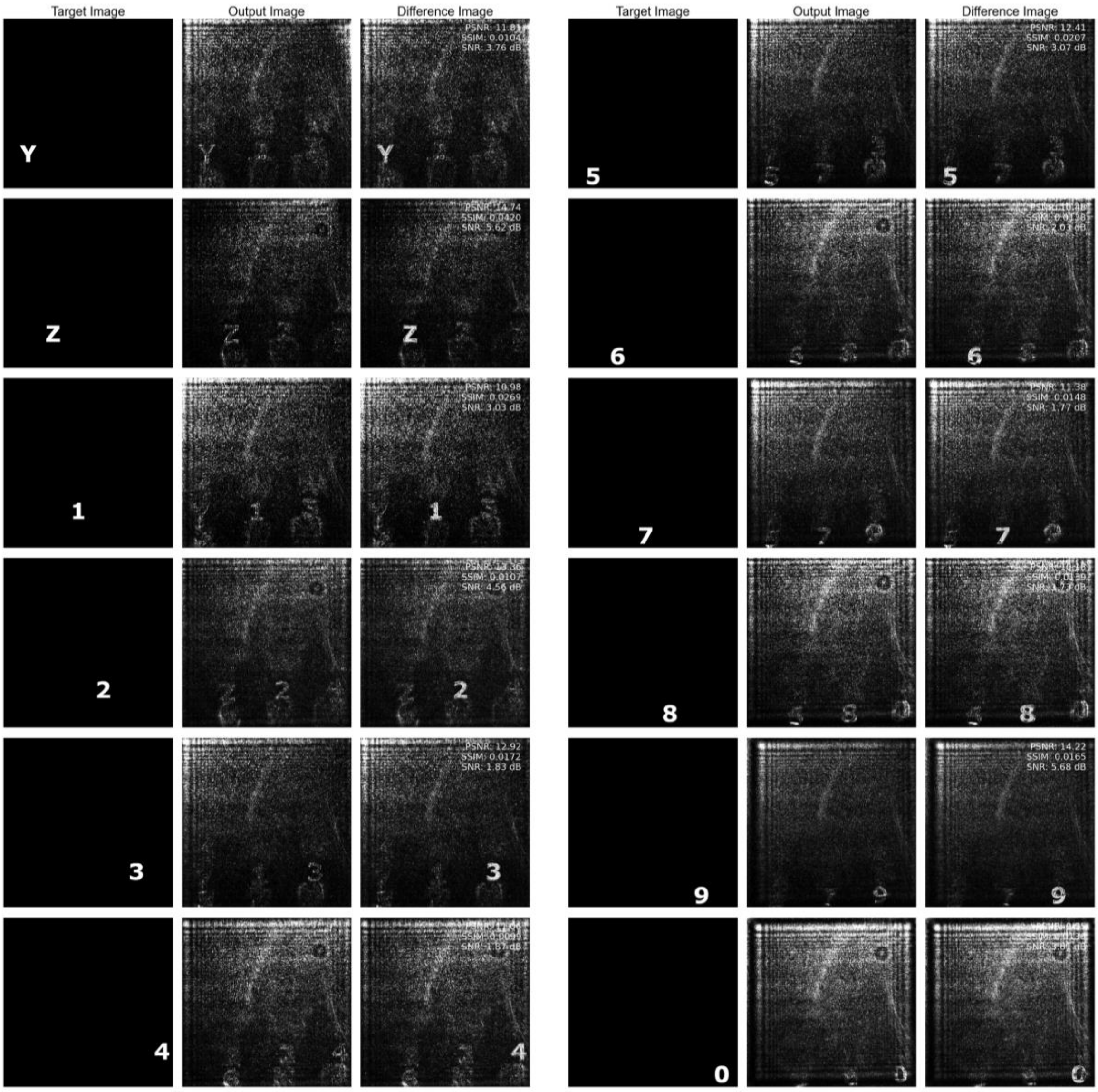

Figure S15. Comparison of PSNR, SSIM, and SNR for the full image in the 36-channel holograms. (a) Target image and the simulated holographic image. (b) Target image, experimentally captured holographic image, and their difference.

Supporting Note 15. Simulation results with varying step sizes

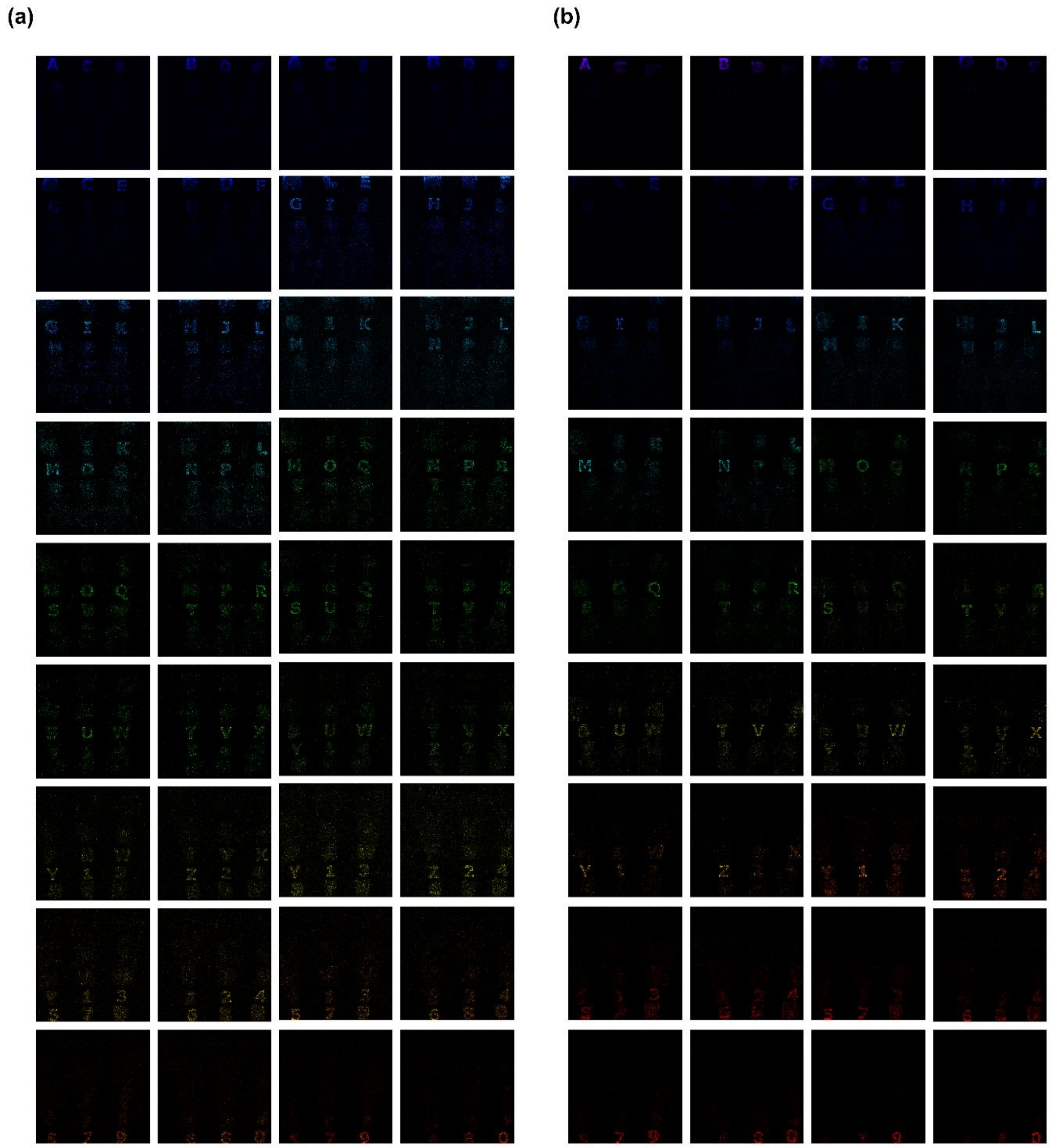

Figure S16. Simulation results for both LCP and RCP polarizations at two different wavelength intervals. (a) Simulations conducted at 10 nm intervals from 470 nm across 18 wavelengths, and (b) simulations at 15 nm intervals starting from 440 nm over 18 wavelengths. The results demonstrate significantly stronger noise when narrower intervals are used, indicating interference between closely spaced wavelengths. Based on this observation, a suitable interval of 20 nm was selected for further experiments.

Supporting Note 16. Simulation results of hologram images based on the number of multiplexing channels and wavelength intervals

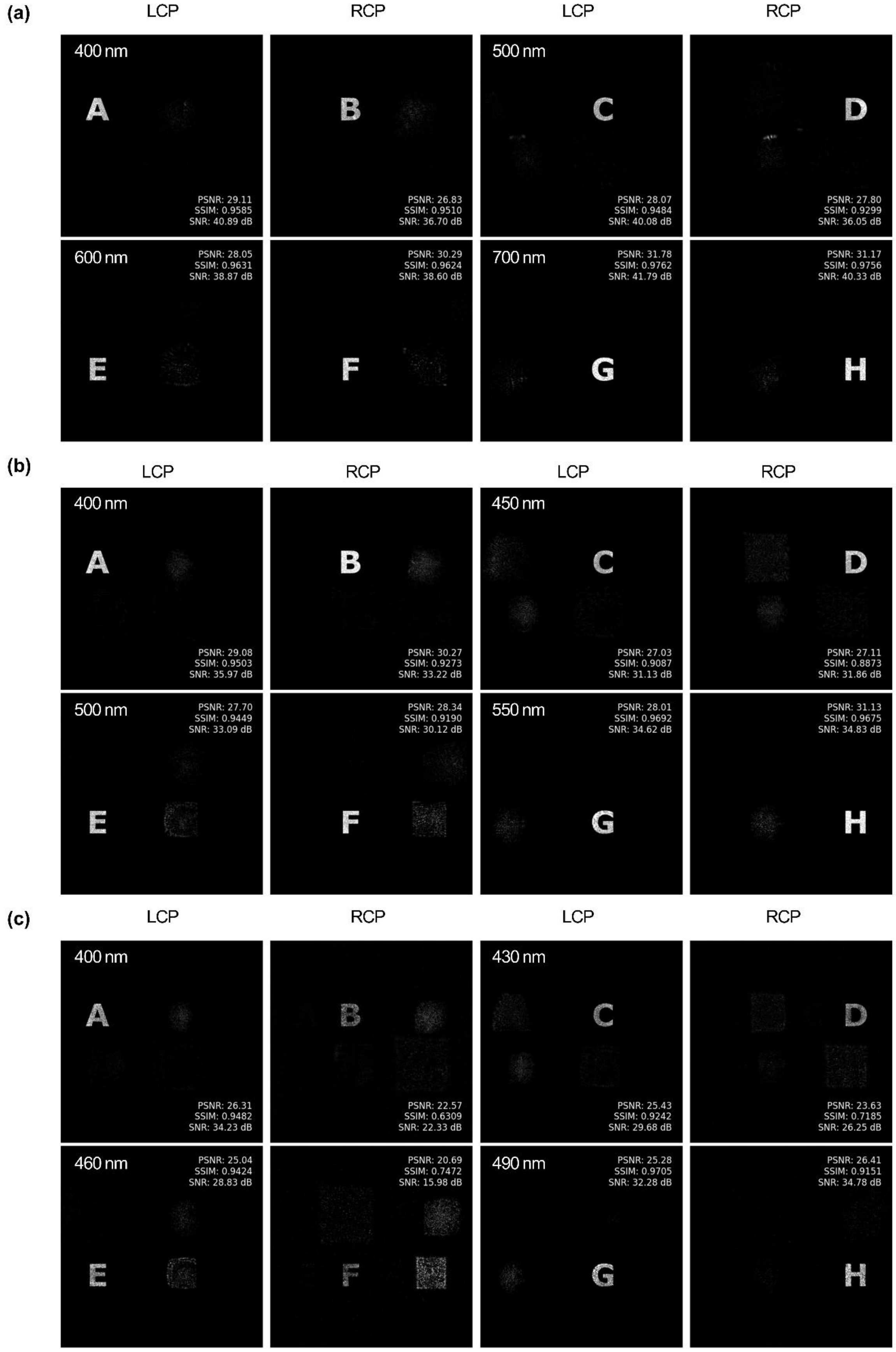

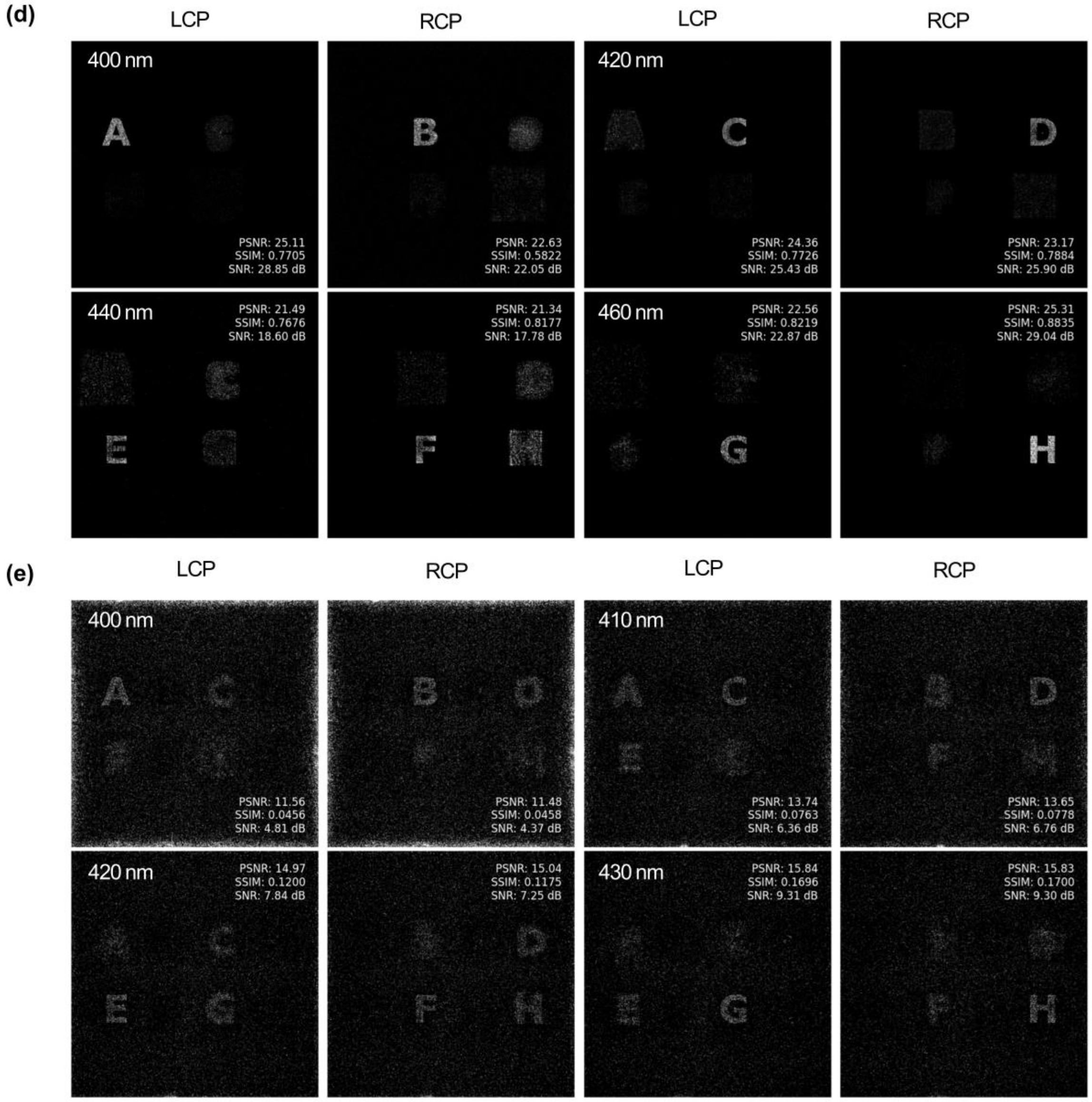

Figure S17. Comparison of PSNR, SSIM, and SNR values for an 8-channel hologram designed with varying wavelength intervals: (a) 100 nm interval, (b) 50 nm interval, (c) 30 nm interval, (d) 20 nm interval, and (e) 10 nm interval.

(a)

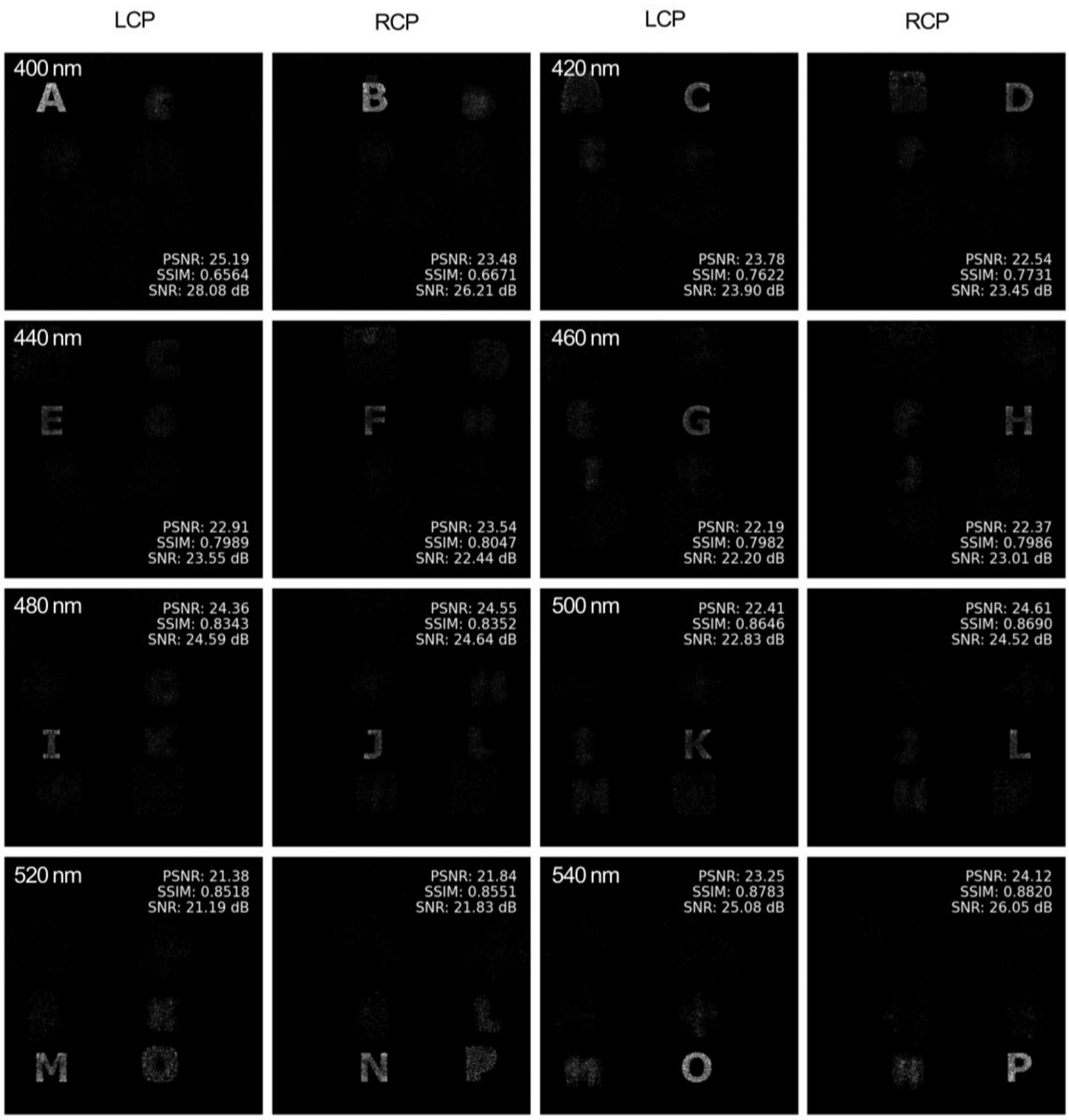
LCP
RCP
LCP
RCP
400 nm
A
PSNR: 25.19
SSIM: 0.6564
SNR: 28.08 dB
B
PSNR: 23.48
SSIM: 0.6671
SNR: 26.21 dB
420 nm
C
PSNR: 23.78
SSIM: 0.7622
SNR: 23.90 dB
D
PSNR: 22.54
SSIM: 0.7731
SNR: 23.45 dB
440 nm
E
PSNR: 22.91
SSIM: 0.7989
SNR: 23.55 dB
F
PSNR: 23.54
SSIM: 0.8047
SNR: 22.44 dB
460 nm
G
PSNR: 22.19
SSIM: 0.7982
SNR: 22.20 dB
H
PSNR: 22.37
SSIM: 0.7986
SNR: 23.01 dB
480 nm
PSNR: 24.36
SSIM: 0.8343
SNR: 24.59 dB
I
PSNR: 24.55
SSIM: 0.8352
SNR: 24.64 dB
J
500 nm
PSNR: 22.41
SSIM: 0.8646
SNR: 22.83 dB
K
PSNR: 24.61
SSIM: 0.8690
SNR: 24.52 dB
L
520 nm
PSNR: 21.38
SSIM: 0.8518
SNR: 21.19 dB
M
PSNR: 21.84
SSIM: 0.8551
SNR: 21.83 dB
N
540 nm
PSNR: 23.25
SSIM: 0.8783
SNR: 25.08 dB
O
PSNR: 24.12
SSIM: 0.8820
SNR: 26.05 dB
P

(b)

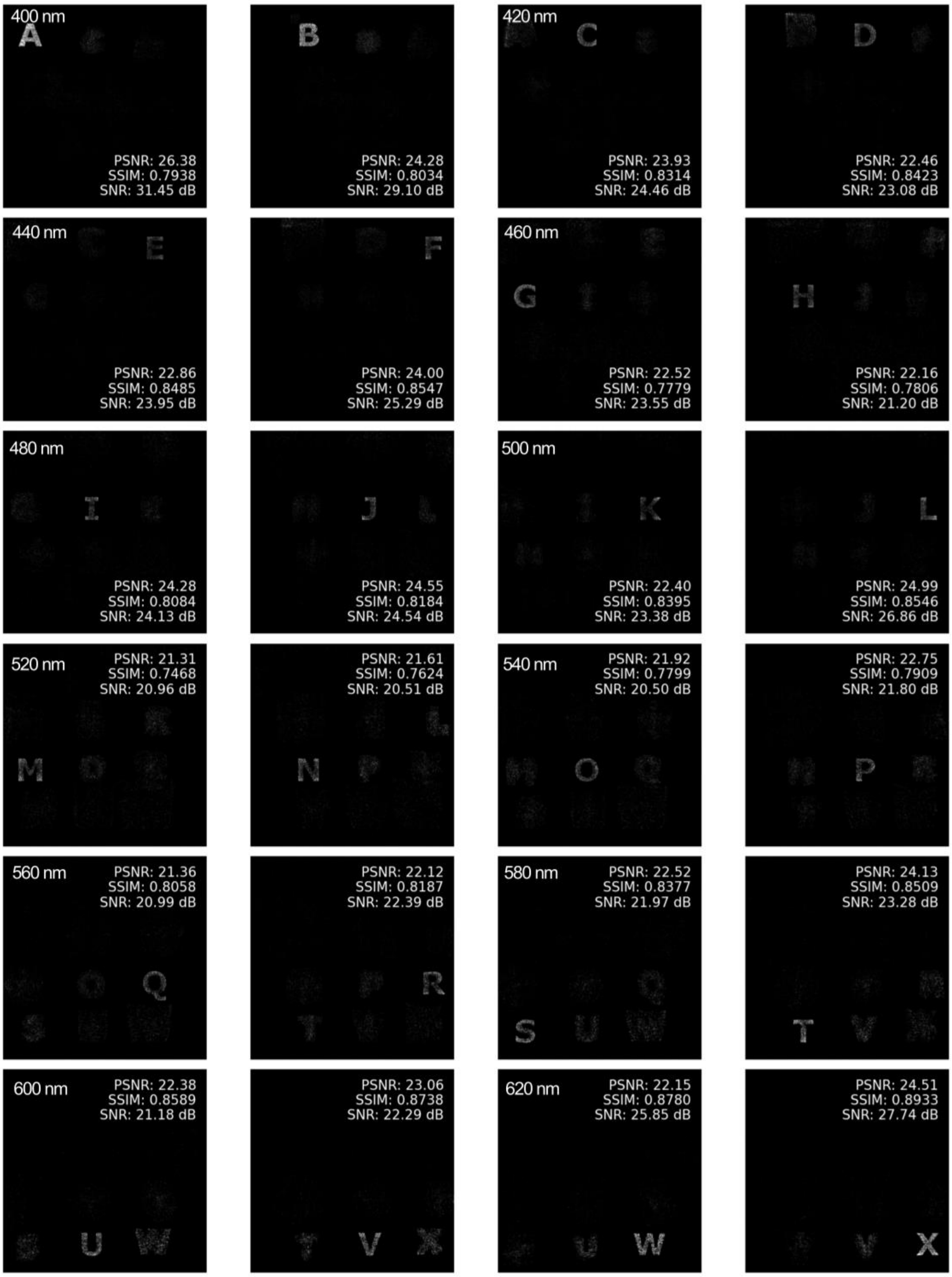

400 nm
PSNR: 26.38 SSIM: 0.7938 SNR: 31.45 dB
PSNR: 24.28 SSIM: 0.8034 SNR: 29.10 dB
420 nm
PSNR: 23.93 SSIM: 0.8314 SNR: 24.46 dB
PSNR: 22.46 SSIM: 0.8423 SNR: 23.08 dB
440 nm
PSNR: 22.86 SSIM: 0.8485 SNR: 23.95 dB
PSNR: 24.00 SSIM: 0.8547 SNR: 25.29 dB
460 nm
PSNR: 22.52 SSIM: 0.7779 SNR: 23.55 dB
PSNR: 22.16 SSIM: 0.7806 SNR: 21.20 dB
480 nm
PSNR: 24.28 SSIM: 0.8084 SNR: 24.13 dB
PSNR: 24.55 SSIM: 0.8184 SNR: 24.54 dB
500 nm
PSNR: 22.40 SSIM: 0.8395 SNR: 23.38 dB
PSNR: 24.99 SSIM: 0.8546 SNR: 26.86 dB
520 nm
PSNR: 21.31 SSIM: 0.7468 SNR: 20.96 dB
PSNR: 21.61 SSIM: 0.7624 SNR: 20.51 dB
540 nm
PSNR: 21.92 SSIM: 0.7799 SNR: 20.50 dB
PSNR: 22.75 SSIM: 0.7909 SNR: 21.80 dB
560 nm
PSNR: 21.36 SSIM: 0.8058 SNR: 20.99 dB
PSNR: 22.12 SSIM: 0.8187 SNR: 22.39 dB
580 nm
PSNR: 22.52 SSIM: 0.8377 SNR: 21.97 dB
PSNR: 24.13 SSIM: 0.8509 SNR: 23.28 dB
600 nm
PSNR: 22.38 SSIM: 0.8589 SNR: 21.18 dB
PSNR: 23.06 SSIM: 0.8738 SNR: 22.29 dB
620 nm
PSNR: 22.15 SSIM: 0.8780 SNR: 25.85 dB
PSNR: 24.51 SSIM: 0.8933 SNR: 27.74 dB

(c)

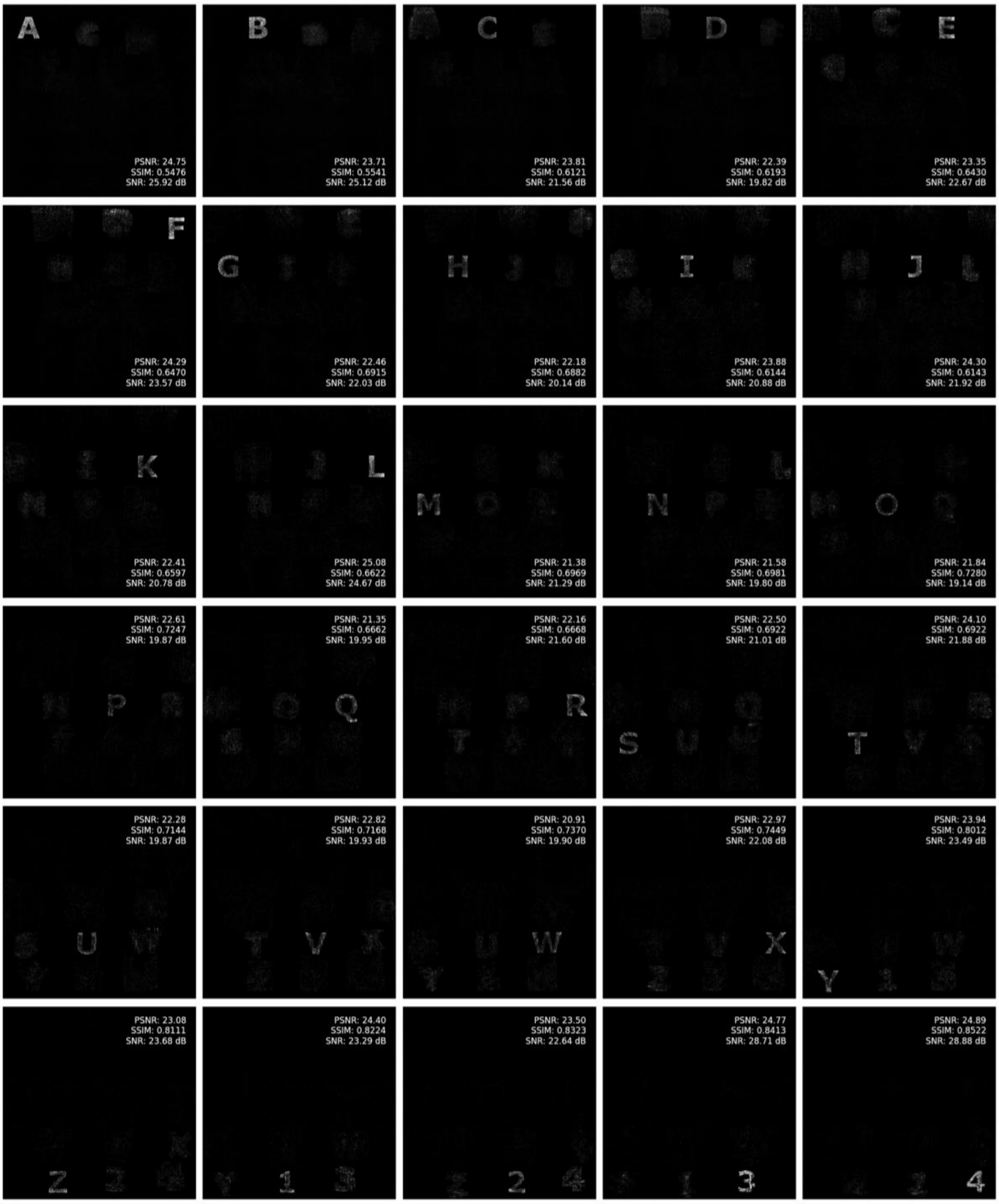
PSNR: 24.75
SSIM: 0.5476
SNR: 25.92 dB
PSNR: 23.71
SSIM: 0.5541
SNR: 25.12 dB
PSNR: 23.81
SSIM: 0.6121
SNR: 21.56 dB
PSNR: 22.39
SSIM: 0.6193
SNR: 19.82 dB
PSNR: 23.35
SSIM: 0.6430
SNR: 22.67 dB
PSNR: 24.29
SSIM: 0.6470
SNR: 23.57 dB
PSNR: 22.46
SSIM: 0.6915
SNR: 22.03 dB
PSNR: 22.18
SSIM: 0.6882
SNR: 20.14 dB
PSNR: 23.88
SSIM: 0.6144
SNR: 20.88 dB
PSNR: 24.30
SSIM: 0.6143
SNR: 21.92 dB
PSNR: 22.41
SSIM: 0.6597
SNR: 20.78 dB
PSNR: 25.08
SSIM: 0.6622
SNR: 24.67 dB
PSNR: 21.38
SSIM: 0.6969
SNR: 21.29 dB
PSNR: 21.58
SSIM: 0.6981
SNR: 19.80 dB
PSNR: 21.84
SSIM: 0.7280
SNR: 19.14 dB
PSNR: 22.61
SSIM: 0.7247
SNR: 19.87 dB
PSNR: 21.35
SSIM: 0.6662
SNR: 19.95 dB
PSNR: 22.16
SSIM: 0.6668
SNR: 21.60 dB
PSNR: 22.50
SSIM: 0.6922
SNR: 21.01 dB
PSNR: 24.10
SSIM: 0.6922
SNR: 21.88 dB
PSNR: 22.28
SSIM: 0.7144
SNR: 19.87 dB
PSNR: 22.82
SSIM: 0.7168
SNR: 19.93 dB
PSNR: 20.91
SSIM: 0.7370
SNR: 19.90 dB
PSNR: 22.97
SSIM: 0.7449
SNR: 22.08 dB
PSNR: 23.94
SSIM: 0.8012
SNR: 23.49 dB
PSNR: 23.08
SSIM: 0.8111
SNR: 23.68 dB
PSNR: 24.40
SSIM: 0.8224
SNR: 23.29 dB
PSNR: 23.50
SSIM: 0.8323
SNR: 22.64 dB
PSNR: 24.77
SSIM: 0.8413
SNR: 28.71 dB
PSNR: 24.89
SSIM: 0.8522
SNR: 28.88 dB

(d)

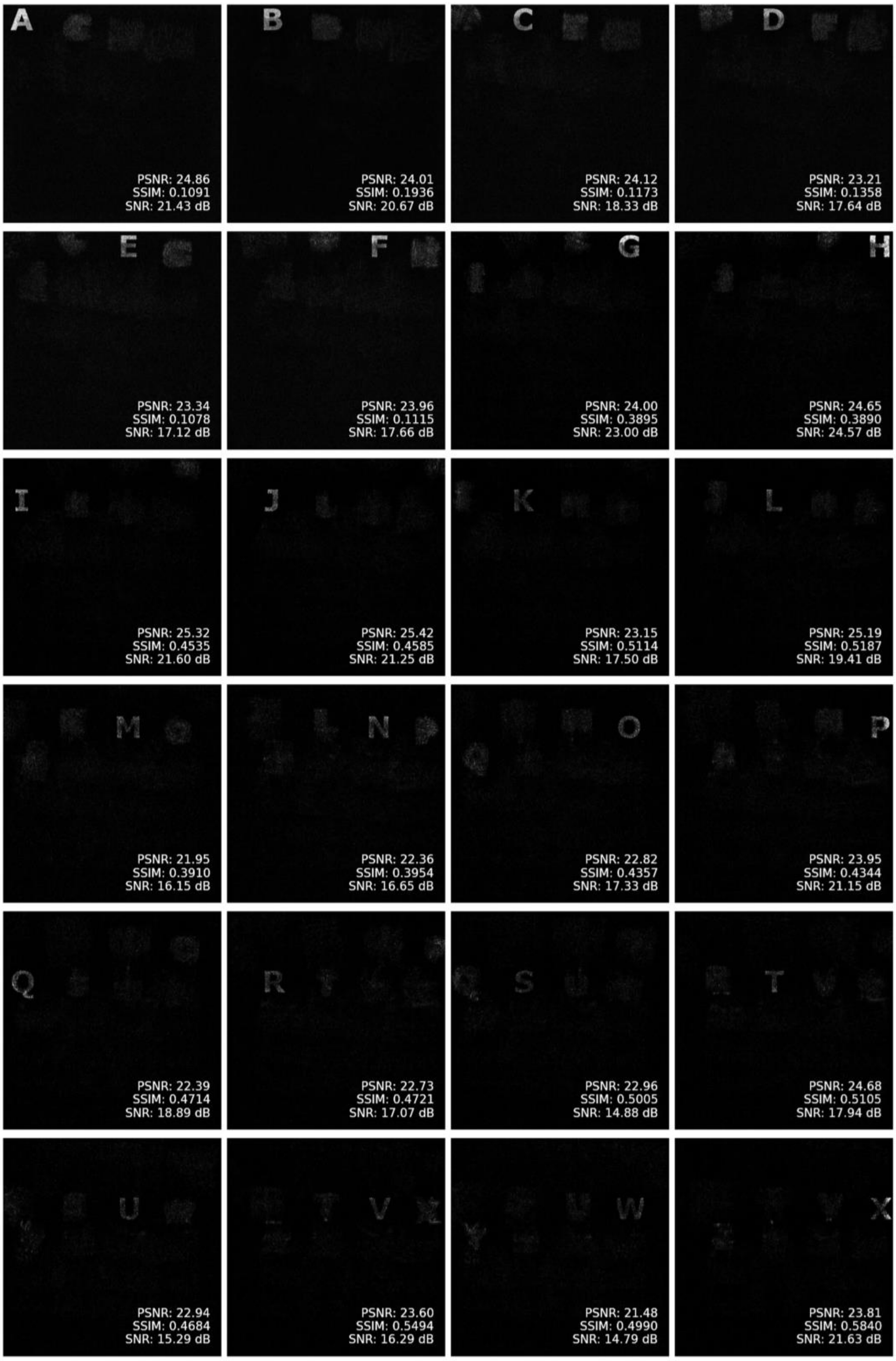
A
PSNR: 24.86
SSIM: 0.1091
SNR: 21.43 dB
B
PSNR: 24.01
SSIM: 0.1936
SNR: 20.67 dB
C
PSNR: 24.12
SSIM: 0.1173
SNR: 18.33 dB
D
PSNR: 23.21
SSIM: 0.1358
SNR: 17.64 dB
E
PSNR: 23.34
SSIM: 0.1078
SNR: 17.12 dB
F
PSNR: 23.96
SSIM: 0.1115
SNR: 17.66 dB
G
PSNR: 24.00
SSIM: 0.3895
SNR: 23.00 dB
H
PSNR: 24.65
SSIM: 0.3890
SNR: 24.57 dB
I
PSNR: 25.32
SSIM: 0.4535
SNR: 21.60 dB
J
PSNR: 25.42
SSIM: 0.4585
SNR: 21.25 dB
K
PSNR: 23.15
SSIM: 0.5114
SNR: 17.50 dB
L
PSNR: 25.19
SSIM: 0.5187
SNR: 19.41 dB
M
PSNR: 21.95
SSIM: 0.3910
SNR: 16.15 dB
N
PSNR: 22.36
SSIM: 0.3954
SNR: 16.65 dB
O
PSNR: 22.82
SSIM: 0.4357
SNR: 17.33 dB
P
PSNR: 23.95
SSIM: 0.4344
SNR: 21.15 dB
Q
PSNR: 22.39
SSIM: 0.4714
SNR: 18.89 dB
R
PSNR: 22.73
SSIM: 0.4721
SNR: 17.07 dB
S
PSNR: 22.96
SSIM: 0.5005
SNR: 14.88 dB
T
PSNR: 24.68
SSIM: 0.5105
SNR: 17.94 dB
U
PSNR: 22.94
SSIM: 0.4684
SNR: 15.29 dB
V
PSNR: 23.60
SSIM: 0.5494
SNR: 16.29 dB
W
PSNR: 21.48
SSIM: 0.4990
SNR: 14.79 dB
X
PSNR: 23.81
SSIM: 0.5840
SNR: 21.63 dB

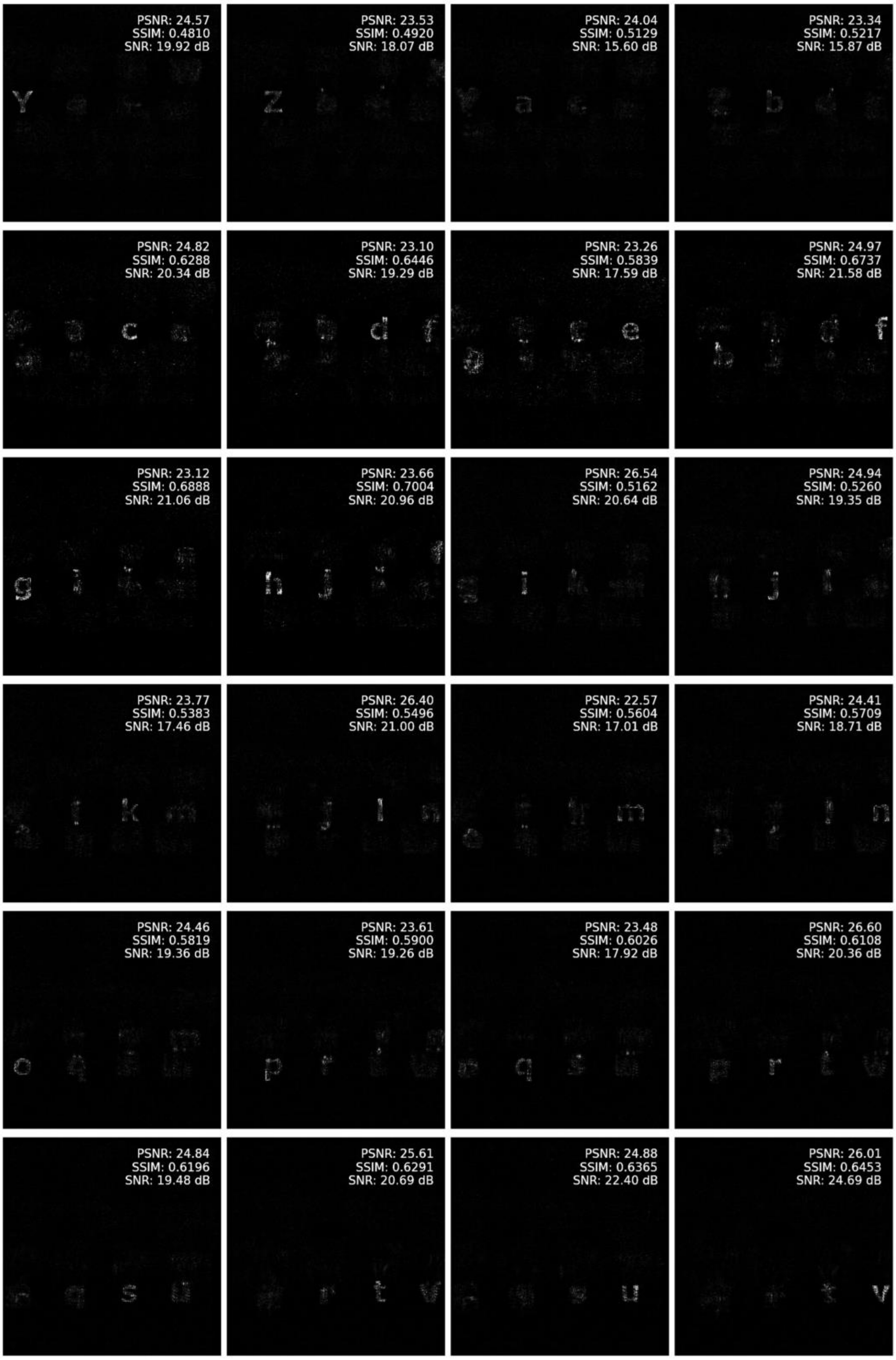

Figure S18. Comparison of PSNR, SSIM, and SNR values for holograms designed with a 20 nm wavelength interval across different numbers of multiplexing channels: (a) 16 channels, (b) 24 channels, (c) 30 channels, and (d) 48 channels

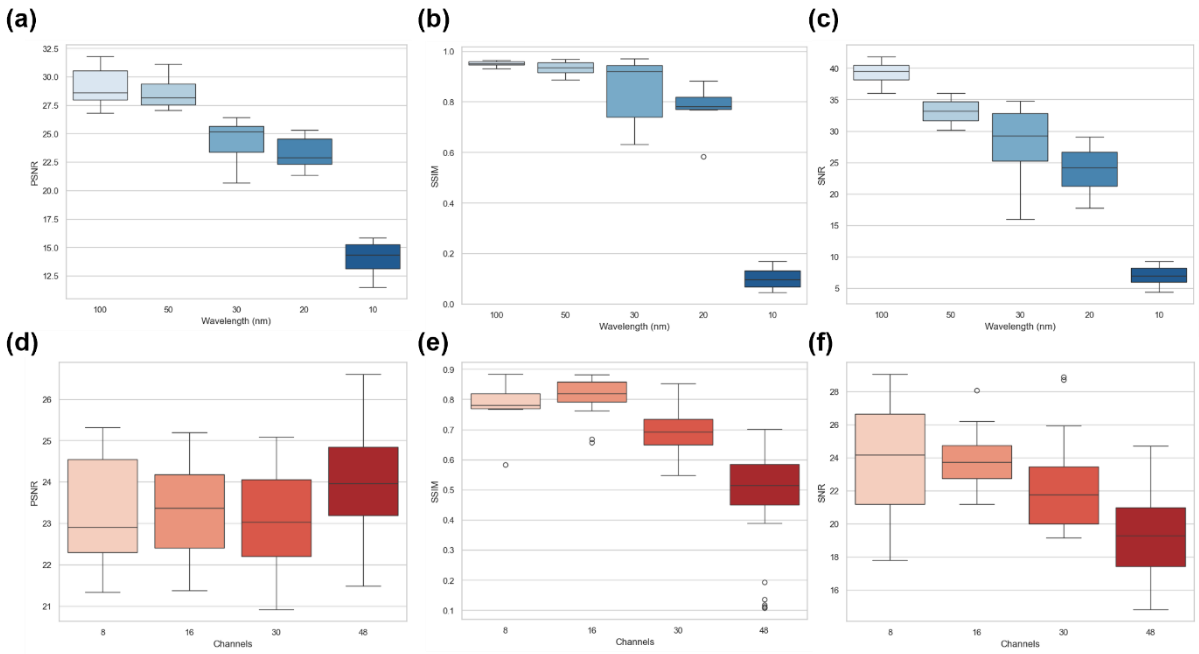

Figure S19. (a-c) PSNR, SSIM, and SNR metrics as a function of wavelength interval (100nm to 10nm). (d-f) PSNR, SSIM, and SNR metrics as a function of the number of channels (8 channels to 48 channels).

Supporting Note 17. Validation of channel scalability in multiplexed hologram design

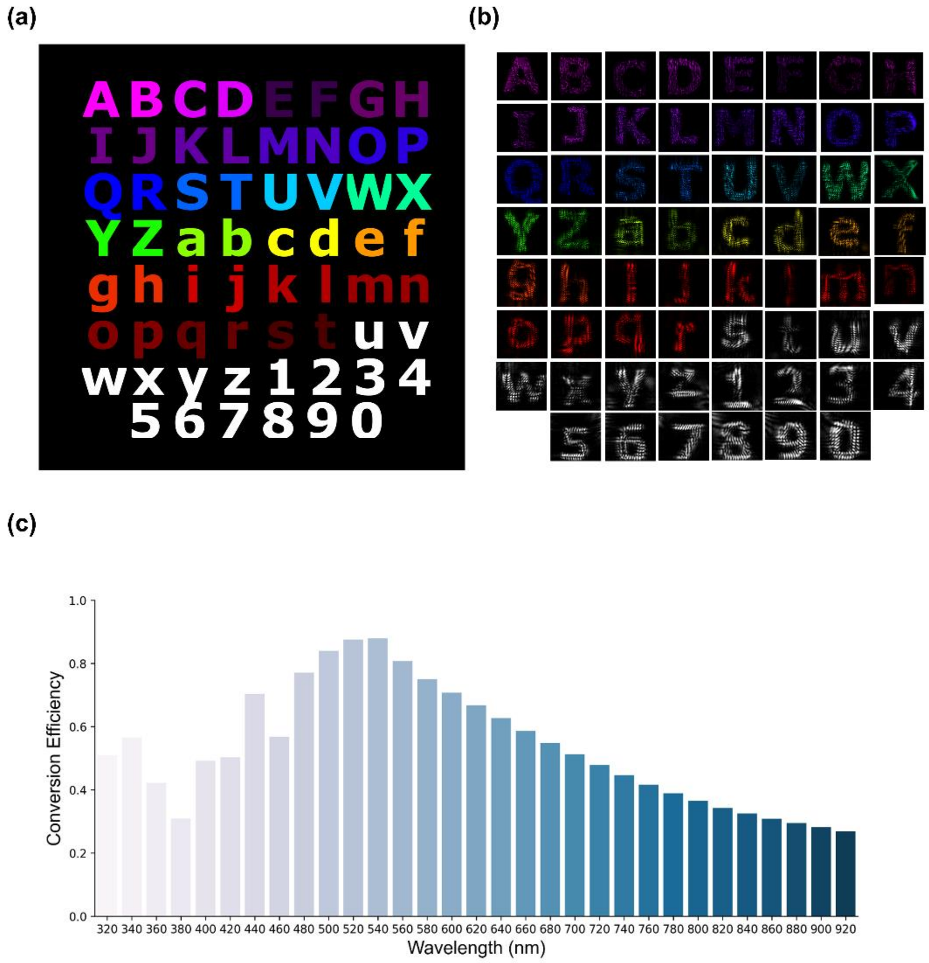

Figure S20. Validation of channel scalability in the design approach, 62 channels are multiplexed, covering both the UV and deep NIR regions. (a) Target image, (b) Simulated image (c) Conversion efficiency data

To validate the scalability of our design approach, we multiplexed a total of 62 images across 31 wavelengths, covering both LCP and RCP, and extending from the ultraviolet (UV) to deeper near-infrared (NIR) regions. To accommodate ultraviolet wavelengths, the pixel pitch is reduced from 450 nm to 350 nm, while all other simulation conditions remained consistent with those outlined in the main text. As the pixel pitch decreases, the allowable width and length of the metaatoms must also be reduced, resulting in a decrease in the overall conversion efficiency compared to previous cases. This effect is especially pronounced in the deeper infrared region, where the refractive index decreases, leading to low conversion efficiency. Consequently, efficiency in experimental implementation may be impacted, a limitation that could be addressed by enhancing the material properties of the metaatoms. For

example, this could be achieved by using a hybrid structure with a material that has an appropriate refractive index. [1,2]

Supporting Note 18. Comparison of high-dimensional multiplexing methods for metasurface holograms

Tabele S1. A comprehensive comparison of existing multiplexing methods for metasurface holograms

Ref.	Number of channels (wavelength / polarization)	Total DOF	Materials	Working wavelength [nm]	Design method	Single cell	Noise-consideration
	1/2	2	TiO2	532	Forward design	X	X
	3/1	3	a-Si	633, 556, 473	Forward design	X	X
	10/1	10	Au	500, 520, 540, 560, 580, 600, 620, 640, 660, 680	Forward design	O	X
	3/2	6	a-Si	633, 532, 488	Forward design	O	X
	3/2	12 (2 image planes)	GaN	639, 556, 478	Inverse design	X	X
	10/1	10	TiO2 PER	420, 450, 480, 510, 540, 570, 600, 640, 680, 720	Inverse design	O	X
	3/1	9 (3 image planes)	TiO2 PER	635, 532, 450	Inverse design	O	X
	1/9	36 (4 image planes)	Au	700	Forward design	X	O
**This work**	**18/2**	**36**	**SiN_x**	**420, 440, 460, 480, 500, 520, 540, 560, 580, 600, 620, 640, 660, 680, 700, 720, 740, 760, 800 (Vis-NIR)**	**Inverse design**	**O**	**O**

This table compares the multiplexing methods for metasurface holograms, emphasizing the advancements introduced in our work. Key aspects include channel capacity (wavelength and polarization), total degrees of freedom, materials used, operating wavelength range, design methodologies (forward vs. inverse design), single-cell implementation, and noise consideration. Our approach stands out with its 36-channel capacity, broader spectral range (420–800 nm), and incorporation of noise-related loss functions to minimize background noise and inter-channel crosstalk. Additionally, the use of a single-phase map for encoding multiple channels simplifies the fabrication process, demonstrating significant progress compared to prior studies.

Supporting Note 19. Number of pixels representing the ROI

Table S2. Number of Pixels Representing the ROI in the 8-Channel Hologram

Image	Dog	Cat	Butterfly	Lion	Bird	Horse	Flamingo	Squirrel
ROI points	25572	29001	33929	31763	16908	23504	15163	23825

Table S3. Number of Pixels Representing the ROI in the 36-Channel Hologram

Image	A	B	C	D	E	F	G	H
ROI points	6422	7637	5495	7286	6279	5089	7182	6965
Image	I	J	K	L	M	N	O	P
ROI points	4526	4223	6801	4211	8705	7881	7264	6167
Image	Q	R	S	T	U	V	W	X
ROI points	8498	7406	6572	4548	6658	5812	10151	6288
Image	Y	Z	1	2	3	4	5	6
ROI points	4848	6096	4358	5444	5674	5563	6090	6351
Image	7	8	9	0				
ROI points	4510	7100	6382	6581				

The total simulation grid consists of 1100 × 1100 pixels, and the ROI corresponds to the area occupied by the target image within this grid.

Supporting Note 20. Measured conversion efficiency of polarization states for the metasurface across wavelengths

Table S4. Measured conversion efficiency

Wavelength [nm]	Conversion efficiency [%]	Wavelength [nm]	Conversion efficiency [%]
420	61.3	620	66.1
440	60.0	640	68.9
460	64.4	660	65.2
480	64.3	680	64.1
500	66.8	700	54.0
520	68.1	720	52.3
540	67.9	740	50.0
560	51.0	760	43.1
580	60.2	800	36.7
600	63.1		

Reference

[1] J. Kim, Y. Kim, W. Kim, D. K. Oh, D. Kang, J. Seong, J. W. Shin, D. Go, C. Park, H. Song, *Mater. Today* **2024**, *73*, 9.

[2] J. Kim, J. Seong, W. Kim, G.-Y. Lee, S. Kim, H. Kim, S.-W. Moon, D. K. Oh, Y. Yang, J. Park, *Nat. Mater.* **2023**, *22*, 474.
